# The Use of Poly-L-Lysine as a Capture Agent to Enhance the Detection of Antinuclear Antibodies by ELISA

**DOI:** 10.1371/journal.pone.0161818

**Published:** 2016-09-09

**Authors:** Nancy A. Stearns, Shuxia Zhou, Michelle Petri, Steven R. Binder, David S. Pisetsky

**Affiliations:** 1 Division of Rheumatology and Immunology, Department of Medicine, Duke University Medical Center, Durham, North Carolina, United States of America; 2 Medical Research Service, VA Medical Center, Durham, North Carolina, United States of America; 3 Bio-Rad Laboratories Clinical Diagnostic Group, 400 Alfred Nobel Drive, Hercules, California, United States of America; 4 Division of Rheumatology, Johns Hopkins University School of Medicine, 1830 East Monument Street, Suite 7500, Baltimore, Maryland, United States of America; Radboud university medical center, NETHERLANDS

## Abstract

Antibodies to nuclear antigens (antinuclear antibodies or ANAs) are the serological hallmark of systemic lupus erythematosus (SLE). These antibodies bind diverse nuclear antigens that include DNA, histones and non-histone proteins as well as complexes of proteins with DNA and RNA. Because of the frequency of ANA expression in SLE, testing is an important component of clinical evaluation as well as determination of eligibility for clinical trials or utilization of certain therapies. Immunofluorescence assays have been commonly used for this purpose although this approach can be limited by issues of throughput, variability and difficulty in determining positivity. ELISA and multiplex assays are also useful approaches although these assays may give an incomplete picture of antibodies present. To develop a sensitive and quantitative ANA assay, we have explored an ELISA platform in which plates are pre-coated with a positively charged nucleic acid binding polymer (NABP) to increase adherence of antigens containing DNA or RNA. As a source of antigens, we have used supernatants of Jurkat cells undergoing apoptosis *in vitro*. As results presented show, a poly-L-lysine (PLL) pre-coat significantly enhances detection of antibodies to DNA as well as antigens such as histones, SSA, SSB and RNP. Comparison of the ELISA assay with the PLL pre-coat with a multiplex assay using the BioPlex^®^ 2200 system indicated good agreement in results for a panel of lupus sera. Together, these studies indicate that a pre-coat with a positively charged polymer can increase the sensitivity of an ANA ELISA using as antigens molecules released from dead and dying cells. This assay platform may facilitate ANA testing by providing an ensemble of antigens more similar in composition and structure with antigens present *in vivo*, with a NABP promoting adherence via charge-charge interactions.

## Introduction

Antibodies to nuclear antigens (antinuclear antibodies or ANA) are the serological hallmark of systemic lupus erythematosus (SLE) and related autoimmune and inflammatory diseases [1,2]. These antibodies bind to a wide variety of nuclear antigens including DNA, histones and non-histone nuclear proteins as well as complexes of proteins with DNA and RNA. Because of the frequency of ANA expression in autoimmune disease, assay of these antibodies has long represented an essential element for clinical evaluation of patients with complex medical illnesses in which lupus, among other diagnoses, is considered [3,4]. In addition, ANA testing is now also used to determine eligibility for entrance into clinical trials as well as the utilization of certain treatments [5,6]. As in the case of many clinical assays, the utility of ANA testing depends upon the balance of sensitivity and specificity and the pre-test likelihood of disease.

With advances in technology, ANA detection has utilized various platforms to enhance performance. Nevertheless, immunofluorescence assays (IFAs) using cultured cells as antigens on slides remain a standard approach [1,7,8]. Such assays, also termed FANAs (fluorescence antibody nuclear antibody assays), while producing valuable data for clinical assessment, have limitations, especially for screening large numbers of samples; performance is time-consuming and involves skill in evaluating fluorescence patterns. Other limitations in the performance of IFAs include variability in results from different kits and uncertainty in titers considered to be clinically significant. Differences among kits may result from conditions for fixation of cells as well as the specificity of detection agents; reader variability can also influence test performance [9–13].

Other approaches for ANA detection have involved enzyme-linked immunosorbent assays (ELISAs) or bead-based multiplex formats [14–22]. In addition to using cell extracts as antigens, these assays have employed purified proteins, cloned products and peptide fragments as antigens, alone or together. Such assays, while well suited for automation and quantitative measurement, can give an incomplete picture of responses especially when only a limited number of proteins is tested [23]. Furthermore, since nuclear antigens are frequently part of multi-component molecular complexes, purified antigens may not display appropriate conformations or higher order structures needed for ANA binding [1].

To develop an immunoassay that can broadly detect ANAs, we have explored a capture ELISA format using a nucleic acid binding polymer (NABP) to increase adherence to the solid phase of antigens that are released from cells during cell death. These polymers have also been termed nucleic acid scavenger polymers [24–28]. NABPs vary in chemical composition and have been designed to bind DNA and RNA avidly on the basis of their charge; these compounds were originally used to create non-viral vectors for transfection or gene transfer. Previous studies demonstrated the potency of NABPs in inhibiting immune responses to DNA and RNA *in vivo* and *in vitro* as well as DNA-anti-DNA interactions *in vitro* [26]. While the use of a NABP would be expected to increase binding of DNA or RNA, the effects on binding of nuclear proteins have not yet been studied although the presence of DNA-protein or RNA-protein complexes could allow enrichment of even protein autoantigens.

To assess the effect of poly-L-lysine (PLL), a representative NABP, as a capture agent for ANA assays, we have performed proof-of-principle experiments using, as an antigen source, supernatants derived from cells undergoing apoptosis. We selected this material since cells undergoing this form of death may be an important source of autoantigens in lupus; also, direct use of molecules released from cells may allow preservation of complexes emerging from the nucleus and provide a more representative set of antigenic structures, including chromatin components that may be modified during apoptosis [29–33]. As results presented herein show, in addition to enriching DNA for immunoassay, PLL can enrich a variety of nuclear antigens and allow sensitive assays for ANA of various specificities. The use of NABP capture thus represents a new format to measure ANA binding to nuclear antigens.

## Materials and Methods

### Materials

PAMAM (PAMAM-G3, polyamidoamine dendrimer, 1,4-diaminobutane core, generation 3.0), HDMBr (hexadimethrine bromide, MW 4,000–6,000); poly-L-lysine (PLL) MW 70-150K, and protamine sulfate were purchased from Sigma Chemical Company (St. Louis, MO). Tetanus toxoid was the kind gift of Richard M. Scearce, Duke University Medical Center, Durham, NC. RPMI, Opti-Mem^™^ medium, gentamicin and Quant-iT^™^PicoGreen^®^dsDNA reagent were purchased from Life Technologies, Carlsbad, CA. Fetal Bovine Serum (FBS) was from Atlanta Biologicals, Norcross, GA. Highly polymerized calf thymus DNA and DNase I were purchased from Worthington Biochemical Corporation, Lakewood, NJ. RNase A was purchased from Teknova, Hollister, CA. All other chemicals were obtained from Sigma Chemical Company unless noted. Jurkat (human T cell lymphoma) cells were obtained from the Duke Cancer Institute Cell Culture Facility.

### Sera and plasma

Plasma from patients with systemic lupus erythematosus (denoted as 1–3) were purchased from Plasma Services Group, Inc. (Southampton, PA). Index plasmas containing antibodies to various autoantigens were purchased from ImmunoVision (Springdale, AR) and were reconstituted according to the supplier’s instructions.

The samples for the comparison of a prototype capture ELISA and the BioPlex^®^ 2200 assay came from a study assessing serological changes during flare in patients from the Hopkins Lupus Cohort. These patients all had an established diagnosis of SLE. Forty-seven of the 48 patients had a positive ANA on at least one time point during their clinical course; there were no data available on one of these patients (619). A positive ANA at the time of entry into the study on flare was not an inclusion criterion. While the study involved samples at three time points for each patient, in this study, only one time point was used [34,35].

### Preparation of nucleosomes

Nucleosomes were prepared from rat liver using a procedure adapted from published methods [36,37]. Concentration of nucleosomes in this paper refers to the concentration of the DNA in the nucleosome preparation as determined from the OD_260_ measured in 10 mM Tris, pH 8, 1 mM EDTA (TE) buffer.

### Preparation of apoptotic cell supernatant (STS supernatant)

Jurkat T cells were grown at 37°C in a humidified atmosphere containing 5% CO_2_ to exponential phase in RPMI with 20 μg/ml gentamicin and 10% FBS. Cells were collected by centrifugation at 500xg for 5min and resuspended at 1x10^7^ cells/ml in Opti-Mem^™^ medium with gentamicin added to 20 μg/ml. Staurosporine (STS) was added to a final concentration of 1 μM and the cells incubated for 24h at 37°C in a humidified atmosphere containing 5% CO_2_. Cells were removed by centrifugation at 500xg for 10 min, and the cleared supernatant transferred to conical tubes and stored at -20°C until use. DNA content was determined using Quant-IT^™^ PicoGreen DNA assay following the supplier’s suggested protocol except that supernatants and standards were diluted in 95% PBS-5% Opti-Mem^™^ medium with 20 μg/ml of gentamicin. Concentrations of STS supernatant in this paper refer to the concentration of the DNA in the supernatant determined in the PicoGreen DNA assay.

### DNase I treatment of apoptotic cell supernatant (STS supernatant)

STS supernatant was prepared as described above and subjected to DNase treatment prior to assay. For this purpose, MgCl_2_ and CaCl_2_ were added to final concentrations of 2.5mM and 0.5mM, respectively, and DNase I in 10mM Tris, pH 7.5, was added to a final concentration of 20 μg/ml (40 Kunitz units/ml). Digestion was performed for 1h at 37°C at which time the reaction was stopped by placement on ice and the addition of EDTA and EGTA to final concentrations of 24 mM and 5 mM, respectively. A volume-adjusted (no DNase) control STS supernatant included all additions except DNase I enzyme; 10mM Tris, pH 7.5 without DNase I enzyme was added instead. The DNased STS-supernatant and volume adjusted control supernatant were assessed by the PicoGreen DNA assay. DNasing resulted in a 78% reduction in the amount of DNA as determined by the PicoGreen assay.

### RNase treatment of apoptotic cell supernatant (STS-supernatant)

Equal volume samples of STS-supernatant were placed into tubes. Bovine pancreatic RNase A was diluted in 10 mM Tris buffer, pH 7.5, to prepare a range of concentrations. An equal volume of each RNase A concentration was added to separate STS-supernatant samples to produce final RNase A concentrations ranging from 1.5 Kunitz units/ml to 0.015 Kunitz units/ml. A volume-adjusted (no RNase A) control sample was prepared by adding the same volume 10 mM Tris buffer, pH 7.5, without RNase A. Digestion was performed for 1h at 37°C. The reaction mix was then frozen for subsequent use.

### ELISA assays

ELISA assays were performed using Immulon 2HB (high binding), flat-bottom, 96-well microtiter plates (Thermo Scientific, Waltham, MA). In these studies, ELISA plates were coated with 100 μl of coating agent (capture polymer or antigen) in buffer overnight at 4°C. Wells were washed 3 times with phosphate-buffered saline, pH 7.4 (PBS), and then blocked with block buffer consisting of 2% bovine serum albumin (BSA), 0.05% Tween 20 in PBS for 2-3h at room temperature (RT; 21–23°C). For polymer capture assays only, wells were next washed 3 times with PBS, and then 100 μl antigen diluted in 0.1% BSA, 0.05% Tween 20 in PBS (ELISA dilution buffer) was added to wells, and the plates incubated 1h at RT.

For all assays, wells were washed 3 times with PBS, 100 μl/well plasma diluted in ELISA dilution buffer was added, and the plates incubated 1h at RT. After incubation, plates were washed 3 times with PBS, 100 μl/well goat anti-human IgG (ɣ chain specific) HRP conjugate diluted 1/1,000 with ELISA dilution buffer was added, and the plates incubated 1h at RT. Wells were washed 3 times with PBS and then incubated with 100 μl of TMB substrate (0.015% 3,3’,5,5’-tetramethylbenzidine dihydrochloride, 0.01% H_2_O_2_ in 0.1M citrate buffer, pH 4) for 30min. The reaction was stopped by adding 100 μl of 2M H_2_SO_4_, and the optical density was read at 450nM with a plate reader.

Unless otherwise noted, plasma dilutions to allow detection of any enhancement by the polymer pre-coat were chosen based on previous titrations performed against antigen coated directly on plates.

### Anti-DNA ELISA

Wells for polymer capture were coated overnight at 4°C with 100 μl/well of 2 μg/ml polymer (PAMAM, HDMBr, poly-L-lysine, or protamine sulfate) in phosphate buffered saline, Ca^2+^ and Mg^2+^ free (PBS-free, Life Technologies). For comparison, other wells were coated overnight at 4°C with 100 μl/well with a range of concentrations of native CT DNA in SSC. The DNA preparation used in these experiments was highly polymerized calf thymus DNA, which was gently resuspended in TE, pH 8, with end-over-end rotation. The DNA concentration was determined by A_260_ spectrophotometer measurement. Since nuclease treatment was not performed to remove any single-stranded regions possibly present, this preparation is denoted as native DNA. After blocking for 2h with 200 μl/well of block buffer, polymer-coated wells were incubated with a range of concentrations of native CT DNA diluted in ELISA dilution buffer. Wells directly coated with CT DNA were allowed to continue blocking in block buffer for this additional hour. Plasma dilutions for this experiment were 1/1,600.

### Anti-nucleosome ELISA

Wells were coated overnight at 4°C with 100 μl/well of PLL diluted to 2 μg/ml in PBS-free. For comparison, other wells were coated overnight at 4°C with 100 μl/well of a range of concentrations of nucleosomes in 20 mM Tris pH 8, 150 mM NaCl. After blocking for 2h with block buffer, wells of the PLL coated plate were incubated with a range of concentrations of nucleosomes diluted in ELISA dilution buffer. Wells coated directly with nucleosomes remained in block buffer for an additional hour of incubation. Antibody was then detected as described above. Plasmas 1 and 2 were used at 1/800; plasma 3 was used at 1/400.

### Apoptotic supernatant ELISA

Wells were coated overnight at 4°C with 100 μl/well of PLL diluted to 2 μg/ml in PBS-free, or with 100 μl/well of a range of concentrations of STS supernatant. After blocking for 2h with block buffer, wells coated with PLL were incubated with a range of concentrations of STS supernatant diluted in ELISA dilution buffer. Wells coated directly with STS supernatant remained in block buffer for this additional hour incubation. Antibody was detected as above. In these experiments, SLE plasmas 1 and 2 were used at 1/1,600; SLE plasma 3 was used at 1/800.

### ANA ELISA

Checkerboard experiments were initially conducted using SLE plasmas and index plasmas all at 1/800, but altering the concentration of PLL coating and the concentration of STS-supernatant (captured by polymer or directly coated). These experiments were performed to select the concentrations for the capture polymer and the STS-supernatant to allow detection of an enhancement of signal provided by polymer capture.

For initial examination, wells were coated overnight at 4°C with 100 μl/well of 500 ng/ml PLL in PBS-free, or with 100 μl/well with a range of concentrations of the STS supernatant. After blocking for 2h with Block buffer, wells coated with PLL were incubated with a range of concentrations of STS supernatant diluted in ELISA dilution buffer. Wells coated directly with STS supernatant remained in block buffer for this additional hour incubation. Antibody was detected as above. Following these experiments, the concentration of the STS supernatant captured or coated directly on plates was held constant at 1,000 ng/ml, and SLE, index and normal plasmas were tested at dilutions ranging from 1/800 to 1/25,600.

### Anti-tetanus toxoid ELISA

Wells were coated overnight at 4°C with 100 μl/well of PLL diluted to 500 ng/ml in PBS-free. For comparison, other wells were coated overnight at 4°C with 100 μl/well with a range of concentrations of tetanus toxoid in PBS-free. After blocking for 2h with block buffer, wells of the PLL coated plate were incubated with a range of concentrations of tetanus toxoid diluted in ELISA dilution buffer. Wells directly coated with tetanus toxoid were allowed to continue incubation in block buffer for this additional hour. Plasmas were used at 1/800.

### Standardization of an ANA ELISA

To determine a cut-off value for the prototype ANA assay, 40 sera (previously identified as lacking ANA activity by a reference laboratory) were tested with Bio-Rad’s ANA EIA Autoimmune Screening Test (96AN) according to the manufacturer’s instructions. This test kit is supplied with a 96-well plate pre-coated with a mixture of dsDNA, histones, SSA (Ro), SSB (La) Sm, SmRNP, Scl-70, Jo-1, centromere and other antigens extracted from the HEp-2 nucleus. Briefly, 100 μl sera diluted 1:40 with supplied sample diluent were incubated in wells pre-coated with antigen. Wells incubated with diluent alone were used as a blanking control. After washing, antibody binding was detected by incubation with the supplied conjugate, followed by the enzyme substrate solution. After incubation for 30 min, the stop color solution was added, and the OD_450_ measured in an EIA plate reader. ANA values were calculated by dividing the OD_450_ measured for each serum by the OD_450_ measured for the calibrator serum. Sera with ANA # > = 1.0 are reported as ANA positive. Sera with ANA # < 1.0 are reported as ANA negative.

### BioPlex^®^ 2200 ANA Screen

48 serum samples collected at the Johns Hopkins Hospital from SLE patients were tested using the Bio-Rad BioPlex^®^ 2200 ANA Screen. This automated, multiplexed technology provides simultaneous detection of 13 autoantibodies. Standards of 6 different concentrations of a calibrated anti-dsDNA serum were tested to allow quantitative determination of anti-dsDNA concentration in International Units (IU). Standards with 4 different concentrations of each of the other antibodies examined in the screen allow semi-quantitative determination of their levels expressed as an antibody index (AI). The testing was conducted with a BioPlex^®^ 2200 ANA screen according to the manufacturer’s methods. Samples with anti-dsDNA antibody determinations of > = 10 IU are considered positive; other ANA antibodies with values of > = 1 AI are considered positive. Sera above the range of the test (>300 IU/ml anti-dsDNA or >8.0 AI other anti-ANA) were diluted and reassayed. The lowest dilution with results in range was used; values reported are those data multiplied by the additional dilution factor.

### Assessment of a prototype ANA kit

These studies were performed at the Clinical Diagnostics Group of the Clinical Immunology Division of Bio-Rad, Hercules, CA. In brief, prototype test kit plates were prepared as follows: ELISA plates (Costar High Binding plate # 2592) were pre-coated with 100 μl/well of PLL diluted to 500 ng/ml in PBS, sealed, and incubated at 4°C overnight. The pre-coating solution was discarded and the plates were washed 3 times with 300 μl/well of PBS. STS-supernatant was prepared at 1 μg/ml in PBS; 100 μl were placed in wells and the plates were sealed and incubated at 4°C overnight. The antigen coating solution was discarded, and the plates were blocked with 200 μl/well with Stabilcoat (SurModics # SC01-1000) at room temperature for 2h. The block solution was discarded and the plates were dried by vacuum and sealed in foil pouches with desiccant until use.

Testing of the 48 SLE patient and the 40 normal sera was conducted using the prototype antigen capture ELISA plates on the Bio-Rad PhD lx automated pipetting workstation using the Bio-Rad ANA Screening Test assay profile modified to include duplicate wells for the blank, calibrator, positive control, negative control and test samples in the assay. Reagents used for the assay included a calibrator (ANA positive serum, #200AN); a positive control (ANA positive serum, R&D # JH-14-13890); a negative control (#200NC); conjugate (Anti-human IgG HRP #6025); sample diluent (#230AD); substrate (#220TM); stop solution (#220SM); and wash buffer (#230AW). ANA values were calculated using the equation: ANA # = additional dilutions factor x OD_sample_ /OD_calibrator_. All samples and standards were diluted 1/40 in dilution buffer for the assay. A cut-off value of 3.9 was established on the basis of the panel of control samples.

## Original Source Data

Original data for all of the figures and tables are provided as supporting information files.

## Results

**Fig 1:**
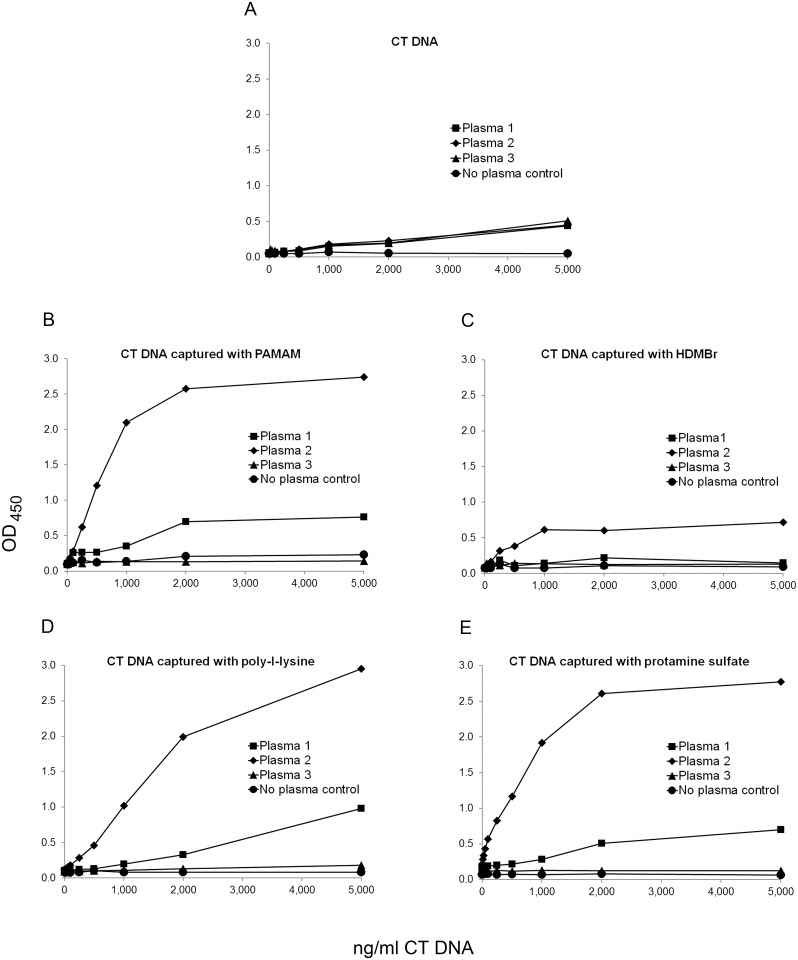
The effect of nucleic acid binding polymers on anti-DNA assays. ELISA plate wells were pre-coated with 2 μg/ml of PAMAM, HDMBr, PLL, or protamine sulfate, and then used to capture native calf thymus (CT) DNA at concentrations from 10 ng/ml to 5,000 ng/ml. Control was incubated with buffer alone. The same concentrations of CT DNA were coated onto plate wells without polymer pre-coat. Binding by antibodies in SLE plasmas 1, 2 and 3 at 1/1,600 dilution was assayed in a standard ELISA assay. Data points represent the average OD_450_ value of two wells except for the data point for 2,000 ng CT DNA coated directly on plate detected with no plasma. That data point represents the OD_450_ value of only one well. Squares show data for plasma 1, diamonds for plasma 2, triangles for plasma 3, and circles for buffer alone. The following are shown: (A) DNA coated directly on plate; (B) DNA with a PAMAM pre-coat; (C) DNA with an HDMBr pre-coat; (D) DNA with a PLL pre-coat; and (E) DNA with a protamine sulfate pre-coat.

In initial experiments, we tested a series of NABPs, including poly-L-lysine (PLL), PAMAM, HDMBr, and protamine sulfate as pre-coats, first assessing effects on DNA antigen. To determine anti-DNA binding, we used patient plasmas previously shown to have high titer anti-DNA activity; while these antibodies bind DNA, they also may contain antibodies to other nuclear molecules. For these experiments, plasmas were titered on microtiter plates coated directly with DNA to determine a dilution that would allow determination of any enhancement. As shown in Fig 1 and S1 Table, pre-coating plates with PAMAM, PLL or protamine sulfate increased levels of antibody binding in 2 of 3 plasmas tested; the effect of the HDMBr was less pronounced.

**Fig 2:**
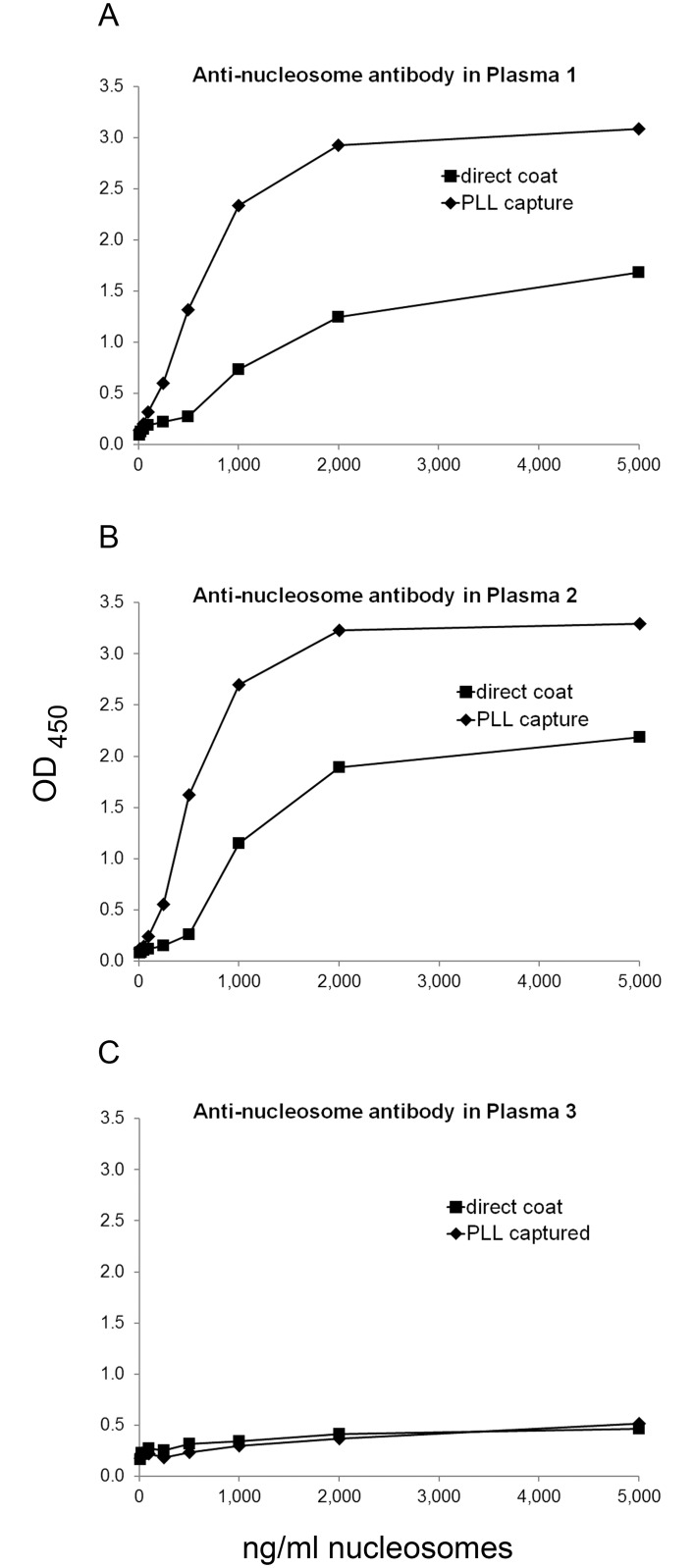
The effect of PLL pre-coat on assays of anti-nucleosome antibodies. ELISA plate wells were pre-coated with 2 μg/ml PLL, and then used to capture nucleosomes at various concentrations (determined from OD_260_ readings) from 10 ng/ml to 5,000 ng/ml, or buffer alone as control. The same concentrations of nucleosomes were directly coated onto plate wells. Binding by antibodies in three SLE plasmas was determined by ELISA. Each point is the average OD_450_ of two wells. Squares show data for nucleosomes directly coated on plate; diamonds show data for nucleosomes captured by polymer. (A) Binding by SLE plasma 1 at 1/800 dilution. (B) Binding by SLE plasma 2 at 1/800 dilution. (C) Binding by SLE plasma 3 at 1/400 dilution.

In subsequent experiments, we focused on PLL of molecular weight 70-150K because of its consistent effects in enhancing antibody binding. Having shown enhancement for purified DNA, we next tested nucleosomes as the antigen. As shown in Fig 2 and S2 Table, a PLL pre-coat augmented binding to nucleosomes; antibodies to nucleosomes commonly co-exist with antibodies to DNA and represent overlapping specificities, although, depending on the preparation, nucleosomes may contain other antigens [38]. In the context of these experiments, these findings suggest that nucleosomes can bind PLL to increase the concentration of antigen on the plate.

**Fig 3:**
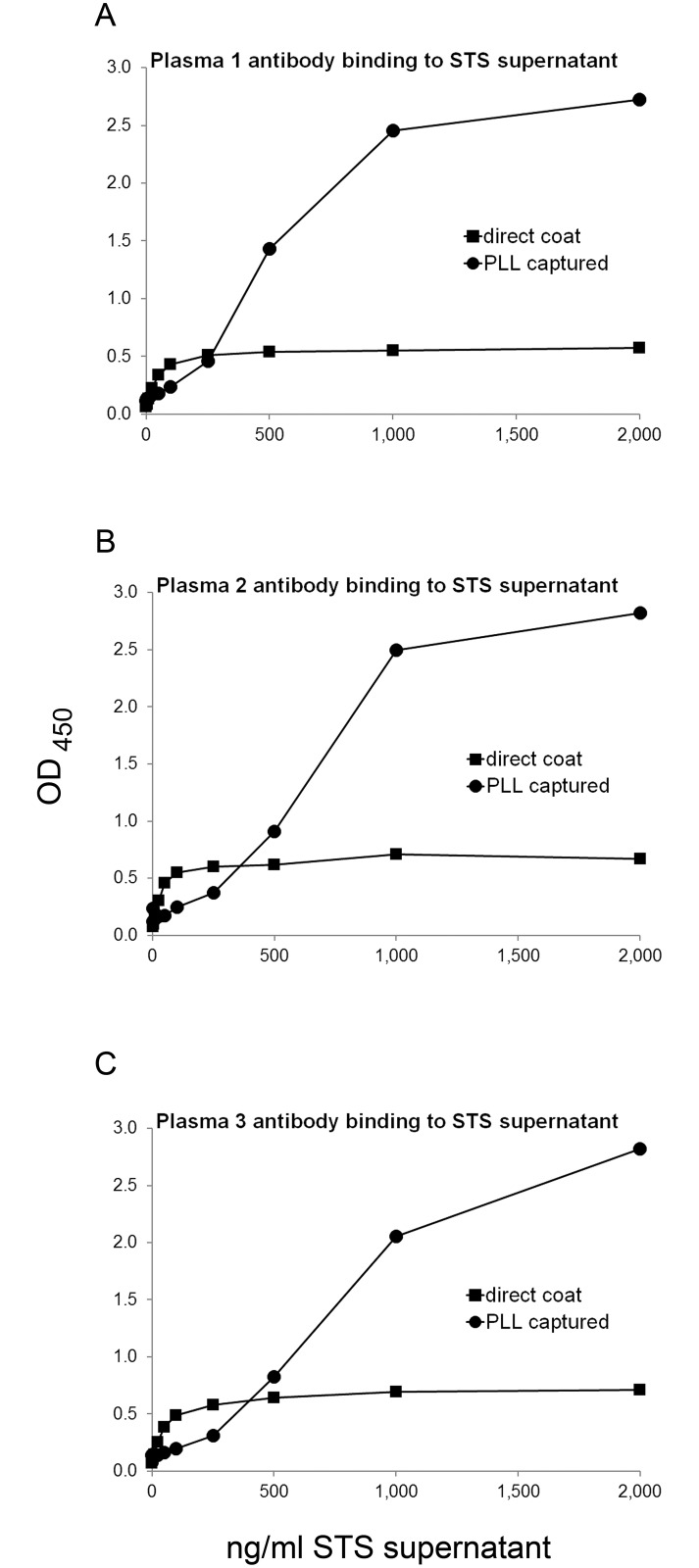
The effect of PLL on ANA assays using supernatant of apoptotic cells as antigen. ELISA plate wells were pre-coated with 2 μg/ml PLL, and then used to capture STS-supernatant at concentrations from 1 ng/ml to 2,000 ng/ml of DNA or buffer alone as control; the concentration refers to that of DNA. The same concentrations of STS-supernatant were coated to plate wells without polymer pre-coat. Binding by antibodies in three SLE plasmas was assayed in a standard ELISA assay. Points shown are the average OD_450_ of two wells. Squares show data for STS-supernatant directly coated on plate; circles show data for STS-supernatant captured by polymer. (A) Binding by SLE plasma 1 at 1/1,600 dilution; (B) Binding by SLE plasma 2 at 1/1,600 dilution; and (C) Binding by SLE plasma 3 at 1/800 dilution.

We extended this approach by testing the antigenic activity of supernatants from apoptotic Jurkat T cells as an antigen. For this purpose, plates pre-coated with PLL were then incubated with the supernatant of Jurkat cells undergoing apoptosis, comparing results with the direct coat of the supernatant to the plates. As results in Fig 3 and S3 Table indicate, the presence of the polymer significantly increased binding of the SLE plasmas.

**Fig 4:**
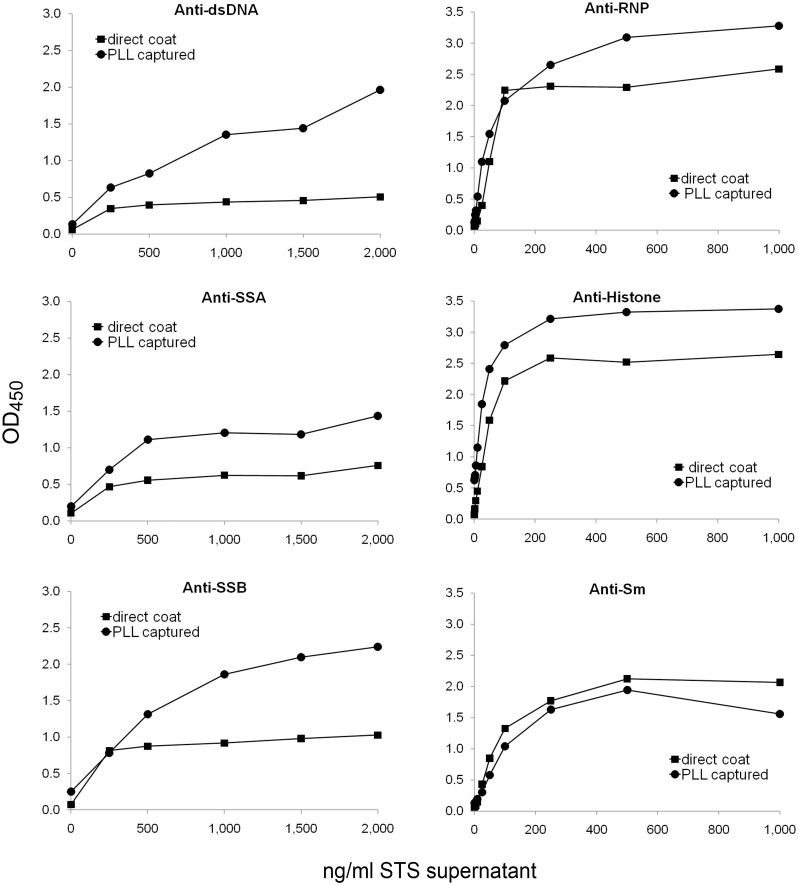
Index plasma binding to STS-supernatant captured with PLL or coated directly onto ELISA plate wells. ELISA plate wells were pre-coated with 500 ng/ml PLL, and then used to capture STS-supernatant at concentrations from 250 ng/ml to 2,000 ng/ml of DNA (left panel) or from 1 ng/ml to 1,000 ng/ml (right panel), or with buffer alone as control. Index plasmas were used at 1/800 dilution in the ELISA. The data points for the anti-dsDNA and anti-SSA index plasmas represent a single well. The data points for anti-RNP, anti-histone, anti-SSB and anti-Sm index plasmas are the average of two wells. Squares show data for STS-supernatant directly coated on the plate; circles show data for STS-supernatant captured by polymer.

To explore the range of specificities detected in the capture assay, we tested the binding of index plasmas for the following antigens: dsDNA, histones, Sm, RNP, SSA, and SSB. Fig 4 and S4 Table presents these results; in these experiments, a single dilution of plasma was tested against varying concentrations of antigen to assess enhancement. As the findings indicate, depending on the specificity, the presence of the PLL pre-coat increased the extent of antibody binding. Thus, for dsDNA, histones, RNP, SSA, and SSB, the pre-coat led to higher levels of antibody binding. In contrast, antibodies to Sm antigen bound well to the antigen preparation irrespective of the presence of the polymer pre-coat. Of note, the pre-coat did not prevent the binding of Sm, suggesting the pre-coat does not non-specifically inhibit antigen binding.

As a nucleic acid binding polymer, PLL can interact with DNA as well as RNA and therefore can potentially capture nuclear antigens that bind either class of polynucleotide; PLL could also capture molecules, that while not directly interacting with a polynucleotide, are part of structures with either DNA or RNA. To determine the nucleic acid dependency on the assay, we treated supernatants with either DNase or RNase and tested the activity of the index plasmas. We compared binding with both direct coating of the antigen preparation as well as the coating of the antigen preparation to the PLL pre-coat. Table 1 and S5 Table presents these results. As these data indicate, DNase could reduce the antigenic activity of the supernatant for at least certain plasmas. For the anti-DNA index plasma, the reduction was 60%. The reduction for the anti-RNP plasma was 51% while the reduction was 14% for anti-Sm. The three SLE plasmas showed inhibition ranging from 46–73%.

**Table 1:** The effect of DNase digestion on the antigenic activity of the supernatant.

		STS			% Reduction
	ELISA	supernatant	OD_450_	OD_450_	of OD_450_
Plasma	format	ng/ml	Control	DNased	With DNase
SLE plasma 1	direct	2,000	0.769	0.781	0
SLE plasma 1	PLL captured	250	0.813	0.417	49
SLE plasma 2	direct	2,000	0.820	0.607	26
SLE plasma 2	PLL captured	250	1.007	0.277	73
SLE plasma 3	direct	2,000	0.485	0.409	16
SLE plasma 3	PLL captured	500	0.871	0.471	46
Anti-SSA	direct	2,000	0.757	0.645	15
Anti-SSA	PLL captured	500	1.112	1.070	4
Anti-SSB	direct	1,500	0.979	0.843	14
Anti-SSB	PLL captured	250	0.878	0.351	60
Anti-dsDNA	direct	2,000	0.515	0.435	15
Anti-dsDNA	PLL captured	1,000	1.055	0.424	60
Anti-histone	direct	250	2.162	2.134	1
Anti-histone	PLL captured	5	0.861	0.743	14
Anti-RNP	direct	500	1.885	1.900	0
Anti-RNP	PLL captured	25	1.100	0.534	51
Anti-Sm	direct	250	1.704	1.730	0
Anti-Sm	PLL captured	100	1.042	0.860	17

The effect of DNase treatment of the STS-supernatant was examined using both the direct coat and the PLL capture ELISA formats. STS-supernatant was DNased for 1h with 40 Kunitz units/ml DNase I. A range of concentrations of untreated STS-supernatant and identically diluted DNased supernatant was coated directly to plates, or captured in wells with PLL, and then examined by ELISA using 1/800 dilution of SLE or index plasmas. STS-supernatant concentration refers to the PicoGreen DNA content of the untreated control. Data shown are for the concentration tested for which the OD_450_ of the untreated STS-supernatant was closest to 1.0. Percent reduction upon DNasing was computed (see text). Cases where signal was higher for DNased supernatant than for untreated supernatant are reported as 0.

**Fig 5:**
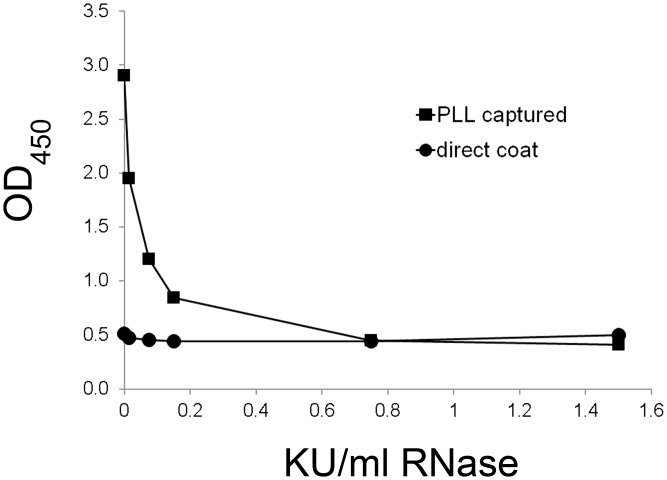
The effect of RNase A treatment of apoptotic supernatant on binding in the ELISA. STS-supernatant was prepared as described and treated with a range of RNase A concentrations from 1.5 Kunitz units/ml to 0.015 Kunitz units/ml or with buffer alone. Samples that were digested and controls were assayed in both a direct coat ELISA and a PLL capture ELISA. SLE plasma 1 was used at 1/800 dilution. Squares show data for PLL capture; circles show data for direct coat ELISA. Data represent 1 well per condition.

The effect of RNase treatment was assessed similarly although these experiments are limited by the capacity of RNase to bind to DNA [39–42]. Thus, any reduction of antigenic activity could result from either RNase digestion of a RNA moiety or inhibition of DNA binding to the PLL because of its binding by RNase. We therefore could assess the effects of RNase at only low enzyme concentrations. Fig 5 and S6 Table shows representative data from one SLE plasma, assessing a range of RNase concentrations. As these experiments suggest, RNase treatment can reduce the antigenic activity of the supernatant although the tendency of RNase to bind to DNA limits a more extensive analysis. Together, these findings indicate some dependency of antigenic activity from the presence of DNA and/or RNA although the extent of the reduction by enzyme treatment varied depending on the plasma and its specificity.

**Fig 6:**
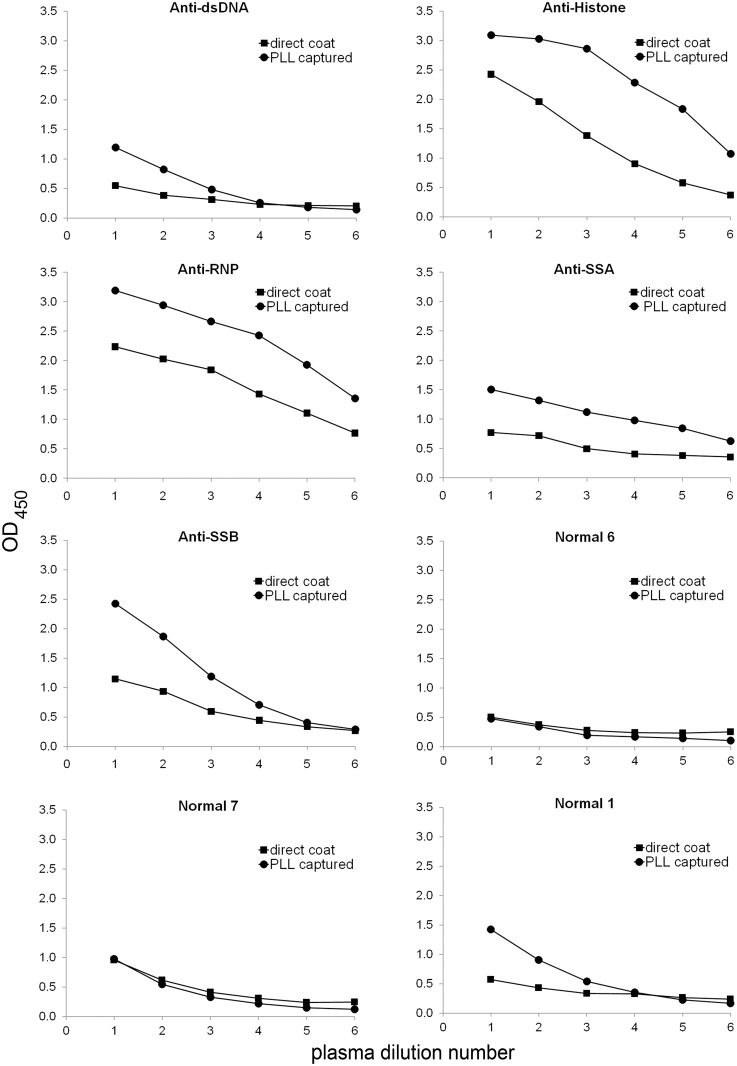
Titration of plasmas against STS-supernatant captured with PLL or coated directly in ELISA plate wells. Index plasmas and normal plasmas were titrated against 1 μg/ml STS-supernatant coated directly in ELISA plate wells or captured onto wells pre-coated with 500 ng/ml PLL. Plasma dilutions ranged from 1/800 to 1/25,600; buffer only was used as control. Squares show data for STS-supernatant directly coated on plate; circles show data for STS-supernatant captured by polymer. Index plasmas used contained the specificities indicated on the charts in the figure.

To assess further any increase in sensitivity afforded by the PLL pre-coat, we titered a series of index and normal plasmas using a single concentration of pre-coat and antigen. Fig 6 and S7 Table presents these results. As these data indicate, for plasmas of each specificity, the presence of a pre-coat led to a higher signal at each dilution compared with the results for the direct coating of the supernatant to the plates. We included normal plasmas to assess any effects of the pre-coat on binding. For two of the three normal plasmas shown, the level of binding was similar with and without the pre-coat. One normal plasma, however, showed increased binding with the pre-coat.

**Fig 7:**
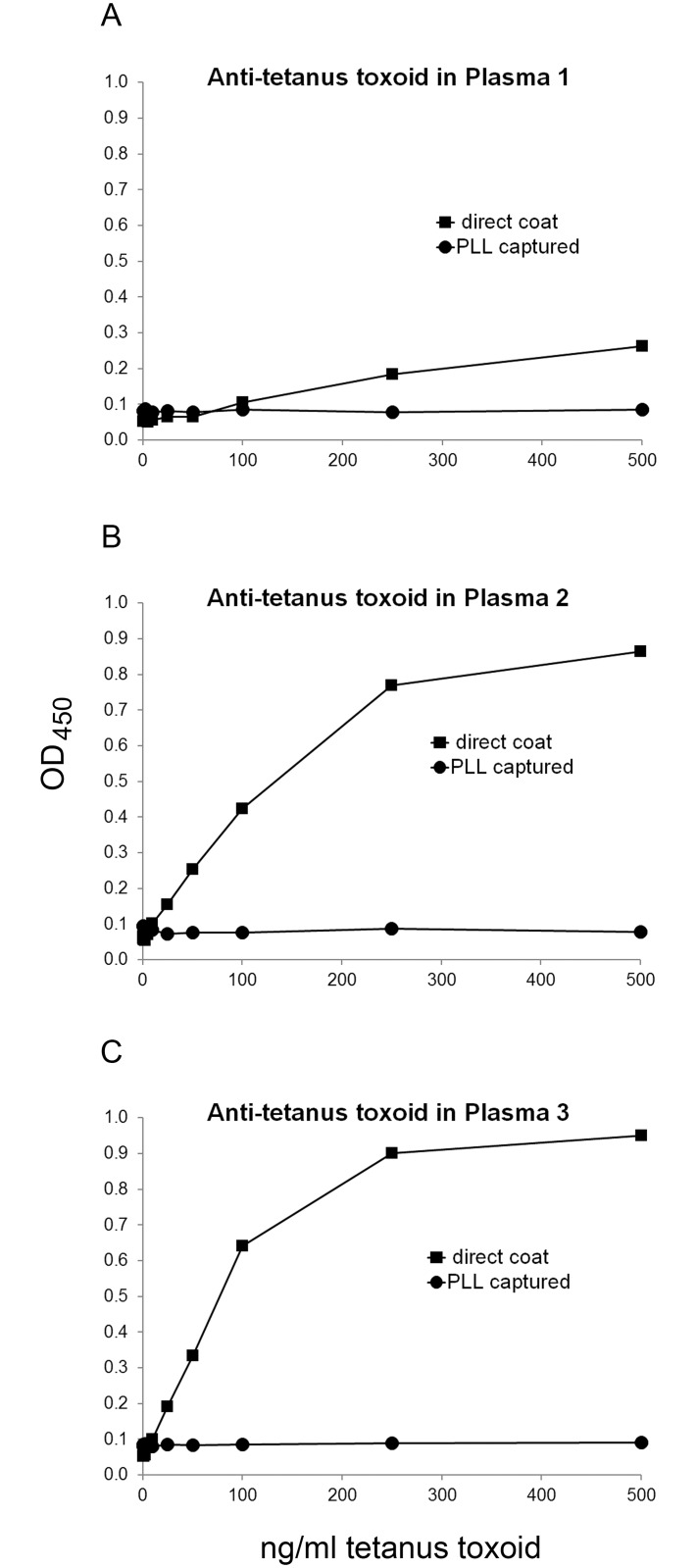
Effect of PLL on assay of antibodies to tetanus toxoid. ELISA plate wells were pre-coated with 500 ng/ml PLL, and then used to capture tetanus toxoid at various concentrations from 25 pg/ml to 500 ng/ml, or buffer alone as control. The same concentrations of tetanus toxoid were coated onto plate wells without the PLL pre-coat. Binding by antibodies in three SLE plasmas was assayed in a standard ELISA assay. Squares show data for direct coat; circles show data for PLL pre-coated plate. All plasmas were tested at 1/800 dilution. (A) Binding by SLE plasma 1. (B) Binding by SLE plasma 2. (C) Binding by SLE plasma 3.

As a control for specificity in these assays, we showed that PLL does not increase the binding of anti-tetanus antibody to tetanus toxoid (Fig 7 and S8 Table). Indeed, the presence of the PLL pre-coat significantly reduced the anti-tetanus antibody activity, likely by preventing binding of the tetanus antigen to the plates. These findings differ from the situation with the nuclear antigens where the presence of the pre-coat did not adversely affect antibody detection with any of the index plasmas.

To determine the relationship between ANA specificity and detection of antibodies by the capture assay, we assayed a panel of lupus sera with a prototype antigen capture assay and compared the results with those of the BioPlex^®^ 2200 multiplex assay. This assay scores individually for 13 self-antigens but, in the context of this study, we are only considering those specificities associated with lupus. Thus, we are not presenting data for Scl-70 or Jo-1. For the capture assay, positivity was determined using an ANA# cut-off value of 3.9 which was determined in the following way: Forty “normal” sera were tested twice with 2 different lots of the Bio-Rad ANA screen EIA (96AN). Thirty-eight (95%) of the 40 sera consistently scored negative and 2 (5%) consistently scored positive. The same 40 sera were tested with the prototype antigen capture assay; ANA# was calculated using the calibrator developed for the kit. Assigning an ANA# cut-off to 3.9 resulted in 95% negative scores and 5% positive scores and thus was chosen as the cut-off for the prototype antigen capture ANA.

As results in Table 2 and S9 Table indicate, there was, in general, good agreement between positivity in the capture assay and the BioPlex^®^ 2200 ANA Test results. Of values positive by the capture assay, all showed positivity for at least one of the assays in the BioPlex^®^ 2200. One sample (0405) had a value of 4.07 in the capture assay which is just above the cut-off. This sample also had values of 1.4 for the SmRNP and RNP A assays in the BioPlex^®^ 2200 assay. There were 6 samples that were positive by BioPlex^®^ 2200 but were below the cut-off by the capture ELISA. Three of these samples (0011, 0216, 1787) were positive for antibodies to Ro60 or SSA-60; serum 0011 also was positive for antibodies to SmRNP. Of sera negative for the capture ELISA, two (0576 and 0735) were positive for anti-dsDNA.

**Table 2:** Comparison of prototype ANA capture assay with BioPlex^®^ 2200 ANA assay data.

		BioPlex^®®^ 2200 ANA Screen Results									
	Capture										
	ANA-EIA	dsDNA	Chrom	Ribo-P	Sm	SmRNP	RNP A	RNP 68	SSB	SSA-52	SSA-60
	ANA #										
Cut-off	3.9	10 IU	1 AI	1 AI	1 AI	1 AI	1 AI	1 AI	1 AI	1 AI	1 AI
Sample											
ID:											
0011	1.90	0.0	0.6	1.0	0.0	**6.6**	**1.8**	0.0	0.0	0.8	**>80**
0014	**32.30**	**19.8**	**>8**	0.2	**>80**	**>80**	**2.2**	0.6	0.2	0.2	1.0
0033	**100.50**	**180.6**	**>8**	**>8**	**4.2**	**>80**	**>8**	0.6	0.0	0.0	0.0
0046	**24.30**	1.0	**>80**	0.2	**>80**	**>80**	0.8	0.8	**>80**	0.6	**>80**
0050	**392.00**	**95.2**	**>800**	0.6	**>800**	**>800**	**>8**	**>80**	0.4	0.0	0.6
0119	**17.85**	**17.2**	**4.4**	0.2	**>8**	**>8**	**4.8**	0.4	0.0	0.0	0.0
0148	0.11	0.4	0.0	0.0	0.0	0.0	0.2	0.0	0.0	0.0	0.0
0167	**35.85**	0.4	**>8**	0.0	0.6	**>80**	**5.0**	**2.0**	0.0	0.0	0.0
0192	**37.85**	**54.4**	**>800**	0.2	0.0	0.6	1.0	0.0	0.0	0.0	**>8**
0216	1.78	0.2	0.2	0.0	0.0	0.0	0.4	0.6	0.0	0.0	**>8**
0221	0.82	2.2	0.2	0.0	0.0	**2.6**	0.6	0.0	0.0	0.0	0.0
0256	**48.50**	**20.6**	**1.4**	0.0	0.0	**1.6**	**1.8**	0.0	0.8	**>8**	**>80**
0259	**15.80**	**26.8**	**4.2**	0.2	**>8**	**>8**	**4.8**	0.4	0.0	0.0	0.0
0272	**578.00**	1.8	**>800**	**>800**	**>800**	**>800**	**>80**	**7.2**	0.4	0.0	**1.8**
0295	**646.00**	1.2	**>80**	**>800**	**>800**	**>800**	**>80**	**2.2**	0.2	0.0	**6.6**
0405	**4.07**	3.4	0.4	0.0	0.2	**1.4**	**1.4**	0.0	0.0	0.0	0.6
0422	0.28	0.4	0.0	0.0	0.0	0.0	0.8	0.0	0.0	0.0	0.0
0428	**17.35**	5.2	**5.4**	**2.4**	0.2	**>80**	**5.8**	0.2	0.0	**>8**	1.0
0456	**150.00**	**>300**	**>80**	1.0	**>800**	**>800**	**>8**	0.0	0.4	0.2	**>8**
0477	**11.45**	3.2	**3.2**	0.0	**>8**	**>8**	0.6	**1.8**	0.0	0.0	0.0
0503	0.80	**14.4**	0.0	0.0	0.0	0.0	0.0	0.0	0.0	0.0	0.0
0510	1.03	5.0	0.2	0.0	0.0	0.0	**2.4**	0.0	0.0	0.0	0.0
0512	1.05	5.6	0.2	0.0	0.0	0.0	0.2	0.0	0.4	0.0	0.0
0534	0.55	0.6	0.0	0.0	0.0	0.0	0.6	0.2	0.0	0.0	0.0
0561	**14.75**	**77.4**	**>8**	0.0	**1.2**	0.6	0.0	0.0	0.0	0.0	0.0
0576	1.23	**263.6**	0.6	0.0	0.0	0.0	0.2	0.0	0.2	0.0	0.2
0582	**614.50**	**63.0**	**>8**	**>8,000**	**>80**	**>8**	**1.6**	0.0	0.2	0.0	**2.2**
0592	**8.80**	3.1	0.4	0.3	0.1	0.1	0.1	0.0	0.9	**>8**	**>80**
0619	**486.50**	**212.2**	**>800**	**>80**	**7.6**	**>8,000**	**>8**	**>800**	0.2	0.0	0.4
0640	2.12	**36.2**	**1.4**	0.0	0.0	0.0	0.0	0.0	0.0	0.0	0.0
0709	0.93	0.8	0.0	0.0	0.0	0.0	**1.4**	0.0	0.0	0.0	0.0
0729	1.12	1.8	0.0	0.0	0.0	0.0	**1.4**	0.0	0.0	0.0	0.0
0735	1.63	**17.4**	**1.2**	0.0	0.0	0.0	**2.2**	0.0	0.0	0.0	0.0
0750	**10.05**	0.2	**1.8**	0.0	0.2	**>80**	0.6	0.6	0.2	0.0	0.2
0784	**5.77**	**22.6**	**4.4**	**4.6**	**4.8**	**3.8**	**1.4**	0.0	0.2	0.0	0.0
0836	0.88	1.6	0.0	0.0	0.0	0.2	0.2	0.0	0.2	0.0	0.0
0935	0.76	0.0	0.0	0.0	0.0	0.0	0.2	0.0	0.2	0.0	0.0
1020	**576.50**	**182.2**	**>800**	**>8**	**4.2**	**>8,000**	**>80**	**>80**	0.2	0.0	**>8**
1028	**16.05**	**21.8**	**4.0**	0.2	0.6	**>80**	**2.0**	**>8**	0.0	0.0	0.0
1076	0.12	0.4	0.0	0.0	0.0	0.0	0.0	0.0	0.4	0.0	0.0
1080	0.96	6.8	0.4	0.0	0.2	0.0	0.2	0.0	0.2	0.0	**2.0**
1309	**154.00**	**>300**	**>800**	**>80**	**>800**	**>80**	**>8**	0.0	**1.6**	0.8	**>8**
1479	**233.00**	0.4	**>80**	0.2	**>80**	**>800**	0.4	**>80**	0.2	0.0	0.2
1679	**44.15**	1.0	**>8**	0.0	**>80**	**>80**	**>8**	**6.0**	0.0	0.0	0.2
1742	**11.25**	0.6	0.2	0.1	0.2	0.1	0.0	0.0	**>80**	**6.5**	**>80**
1764	1.00	0.2	0.0	0.0	0.0	0.0	0.0	0.0	0.0	0.0	0.0
1787	1.03	2.0	0.2	0.0	0.0	0.0	**2.6**	0.0	0.0	0.8	**>8**
1842	**35.15**	**167.2**	**>8**	**5.8**	**>8**	**3.6**	**6.4**	0.0	0.4	**>80**	**>8**

Sera from 48 SLE patients were tested in both the prototype ANA capture ELISA and the BioPlex^®^ 2200 ANA Screen as described in the text. Data in bold type represent ANA positive test results. ANA numbers for the prototype ANA capture assay were calculated as ANA# = additional dilution factor above 1/40 X OD_sample_ /OD_calibrator_. BioPlex^®^ 2200 ANA Screen testing was conducted according to the manufacturer’s protocol. Results of screening for antibodies to double stranded DNA (dsDNA); chromatin (Chrom); riboprotein (Ribo-P); Smith antigen (Sm); Smith antigen + ribonucleoprotein (SmRNP); RNP A; RNP 68; La/SSB (SSB); Ro/SSA52 (SSA-52); Ro/ SSA60 (SSA-60) are reported. Standards of 6 different concentrations of a calibrated anti-dsDNA serum were tested to allow quantitative determination of anti-dsDNA concentration in International Units (IU); sera with anti-dsDNA antibody determinations of > = 10 IU are considered positive. Standards with 4 different concentrations of each of the other antibodies examined in the screen allow semi-quantitative determination of their levels expressed as an antibody index (AI). Sera with AI values of > = 1 are considered positive. Sera above the range of the test (>300 IU/ml anti-dsDNA or >8.0 AI other anti-ANA) were diluted beyond the base dilution (of 1/40) and re-assayed. The lowest dilution with results in range was used; values reported are those data multiplied by the additional dilution factor.

A correlation analysis was performed to assess the association between the prototype ANA capture and the BioPlex^®^ 2200 ANA assays. Using the pre-determined cutoff values, data for each sample was transformed to positive (above the threshold) or negative (below the threshold). For the BioPlex^®^ 2200 ANA assay, a sample was categorized as positive if any of the 10 antigens tested positive. Using GraphPad, a Spearman correlation was performed, with statistical significance set at p ≤ 0.05. The Spearman correlation coefficient between the two assays was 0.568, with p < 0.0003. These results indicate that the two assays are highly correlated, and the association is highly significant.

The agreement between results of the ELISA and determination of anti-chromatin and anti-DNA chromatin is notable. Thus, among all the sera that were positive for anti-DNA or anti-chromatin, these samples were also positive in the capture ELISA with two exceptions. These samples (0640 and 0735) had low values in anti-chromatin assay (1.4 AI and 1.2 AI). These sera, however, had more robust levels of anti-DNA (36.2 AI and 17.4 AI). In this series, 4 sera had significant anti-chromatin responses (0167, 0477, 1479, 1679) without comparable positivity for anti-DNA. Therefore, the agreement of the capture ELISA and the anti-DNA and anti-chromatin assays in the BioPlex^®^ 2200 indicate that both technologies can effectively detect antibodies in this group of antigens. Of note, studies suggest that the frequency of anti-chromatin (an antigen consisting of histones and DNA) is greater than the frequency antibodies to DNA in SLE [43].

Along with anti-DNA, anti-Sm is also a serological criterion in the classification of patients with SLE. It is notable that all sera positive for anti-Sm by the BioPlex^®^ 2200 assay were also positive by the capture ELISA. Indeed, the highest values in the capture ELISA were observed in sera that were positive for anti-Sm or anti-SmRNP. Together, these findings suggest that a capture ELISA, when prepared using an apoptotic supernatant, can detect antibodies directed to most of the commonly targeted autoantibodies found in lupus sera, including those that have significance as classification criteria.

## Discussion

Results presented herein describe a new approach to ANA detection utilizing poly-L-lysine (PLL), a nucleic acid-binding protein (NABP), to promote the binding of nuclear antigens to the solid phase and the use of supernatants of dead and dying cells as a source of antigen material. The advantage of such material may relate to the retention of molecular associations important for antigenicity. Thus, we have shown that pre-coating with PLL can produce assays with enhanced ability to detect antibodies to certain ANA specificities, with control experiments using DNA and nucleosomes as antigens showing enhanced binding by lupus plasmas. Other studies using index plasmas showed increased binding of anti-histone, anti-SSA, anti-SSB, and anti-RNP antibodies; since histones are components of nucleosomes and chromatin, increased binding of plasma characterized as anti-nucleosome or anti-chromatin would also be expected. In contrast, the pre-coat had less effect on the binding of antibodies to Sm. These results suggest that nuclear antigens released from dead and dying cells can be associated with DNA/RNA in a physical-chemical form that allows enrichment on the PLL support.

As shown previously, NABPs can bind to both DNA and RNA in *in vivo* and *in vitro* systems to prevent immune cell activation, presumably by sequestering these molecules and blocking interaction with toll-like receptors (TLR) and other non-TLR nucleic acid sensors [24–28]. Other studies have demonstrated that NABPs can bind to DNA to inhibit the interaction with anti-DNA antibodies as well as displace anti-DNA from preformed complexes [26]. Since NABPs bind strongly to nucleic acids, we wondered whether DNA or RNA released from cells had sufficiently long stretches of charge to form a stable interaction with a NABP and allow binding along with any attached proteins that may be antigenic. Not unexpectedly, the presence of the polymer pre-coat increased the sensitivity of anti-DNA assays with purified DNA as antigen. Notably, we showed that a PLL pre-coat enhanced antibody detection using purified nucleosomes and supernatant preparations as antigens. The enhancement of binding of supernatant antigens was observed with a variety of index plasmas of different ANA specificities, suggesting that antigens released from dying cells have accessible stretches of DNA or RNA to allow polymer binding; alternatively, these proteins may have regions of negative charge that allows interaction with PLL.

While the presence of at least some regions of “free DNA” in the cell is likely, the extent of such regions is unknown [44–46]. Our findings suggest the nuclear material released from apoptotic cells contains DNA in a free or accessible form to allow interaction with the polymer; this approach may also enrich for RNA as well as proteins associated with RNA such as the ribosomal P protein. This material appears to be present reproducibly as indicated by the performance of the assay over time. Relevant to this issue is the binding of nucleosomes to the polymers. While a close association of DNA with histones could limit polymer binding, our results with purified nucleosomes suggest that, even in this structure, DNA is accessible and binds to polymer, thus leading to a greater concentration of antigen on the plate. The binding to PLL would not be expected to perturb the structure of nucleosomes since the binding occurs under ordinary salt conditions.

The results with antibodies to RNA binding proteins (RBPs) produced a somewhat different picture since we showed enhancement with antibodies to SSA, SSB and RNP but not Sm. These findings suggest that, as in the case of nucleosomes, certain RBPs can have sufficient regions of accessible RNA to bind to polymers. It is possible, however, that the RBPs have some association with DNA perhaps as part of complexes or microparticles which have surface DNA. We have shown previously that microparticles contain Sm antigen as shown by flow cytometry; other investigators have shown the presence of SSB in microparticles [47–49].

The studies on the effects of DNase and RNase provide some insight into these issues. For three lupus plasmas, DNase treatment led to a reduction in antigenic activity of the supernatant bound to the PLL pre-coat; this reduction was greater than that observed when the supernatant was bound directly to the plate although the overall binding was much greater with the PLL pre-coat. For the index plasmas, the reduction of activity was variable. The experiments with RNase treatment are more difficult to interpret since RNase can bind DNA and therefore also inhibit interaction of DNA with the PLL pre-coat [39–42]. Despite these limitations, we were able to show that, with one SLE plasma, RNase treatment at low concentration could reduce antigenic activity of the supernatant bound to the PLL pre-coat.

A further issue with the experiments with both DNase and RNase relates to the extent of digestion of either DNA or RNA in the setting of the supernatant where the concentration of other proteins, including molecules that bind to DNA and RNA and prevent access of the enzymes, is high. With DNase, for example, we achieved only a 73% reduction in PicoGreen binding even with the maximal concentrations of the enzyme used. Nevertheless, despite these limitations, the current results are consistent with a role of both DNA and RNA in polymer binding of autoantigens although direct binding of some nuclear autoantigens may also occur.

Other aspects of this study bear comment. The first concerns the use of supernatants as a source of nuclear antigens for solid phase assays. While the source of nuclear molecules that impinge on the immune system in lupus is not known, studies using *in vivo* and *in vitro* systems suggest that apoptotic cells can release autoantigens to drive autoreactivity. The expression of antibodies to histones that have been modified during apoptosis provides additional evidence of the role of this death form in generating extracellular nuclear autoantigens [32,33]. Such autoantigenic material can also form immune complexes that deposit in the tissue or drive cytokine production [50]. These findings do not exclude a role for other death forms (e.g., NETosis, necroptosis) in releasing autoantigens that promote pathogenesis.

For these experiments, we have used supernatants of Jurkat T cells that have undergone apoptosis by treatment with staurosporine, a broad protein kinase inhibitor. We have shown previously that these cells release, in a time-dependent way, DNA as well as HMGB1, a non-histone protein with alarmin activity [51,52]. For the immunoassays, we used supernatants from cells cultured for 24 hours at a stage that can be termed secondary necrosis, since cells at that time point stain with both annexin V and propidium iodide (data not shown). Preliminary studies indicate that supernatants from cells undergoing primary necrosis by freeze-thaw can also be a source of nuclear antigens for assays of this kind. Thus, it is possible that the use of other antigen sources (e.g., cell extracts, supernatants of different cell lines) could alter the frequency of positive values in either lupus plasmas or plasmas from normal individuals.

In this regard, cells undergoing apoptosis may release cytoplasmic as well as nuclear molecules; mitochondria may be a source of these molecules, including DNA [53,54]. As our results show, this assay can detect antibodies to the ribosomal P antigens; ribosomal P protein is a component of the ribosome, a cytoplasmic structure. It is therefore possible that the PLL can capture other cytoplasmic molecules that could represent autoantigens. Such molecules may include mitochondrial DNA which should be bound by anti-DNA autoantibodies. Studies are in progress to determine the range of other autoantibodies (e.g., anti-mitochondrial antibodies) that can be detected in this assay.

Another issue concerns the use of a NABP as a pre-coat for the development of solid phase assays. Such a “capture” approach, which can be performed with specific antibodies, can increase solid phase adherence of an antigen and has previously been utilized extensively with assays of anti-DNA antibodies [55–59]. Depending on the ELISA plates, some DNA preparations, especially those in the double stranded conformation, may not bind well; a pre-coat of PLL or protamine sulfate increases reproducibility and sensitivity. In our study, while a pre-coat of PLL augmented DNA binding and antigenicity, it also affected the antigenic activity of some other, but not all, nuclear molecules.

Our study has a number of limitations. Thus, we have explored this approach in proof-of-principle experiments, focusing primarily on lupus. While preliminary studies with index plasmas (i.e., anti-Scl-70) suggest utility in screening for other rheumatic and autoimmune diseases, we have not specifically analyzed this issue. Such studies are in progress. We also do not know the ability of this assay to detect antibodies binding to other nuclear antigens such as HMGB1 which can interact with DNA and histones [60]. Another limitation relates to the responses of patients with lupus since we observed that sera of many patients had low values in the capture ELISA; these sera also had low values in the BioPlex^®^ 2200 assay. While expression of ANA has been considered almost invariable in this disease, increasing data indicate that changes in serology can occur over time, especially with treatment, and that patients with a well-established diagnosis may lack ANA positivity [61–67].

By design, this study provides data on feasibility and does not address the performance of this assay in the clinical setting. While the increased antibody detection afforded by the presence of the pre-coat would suggest increased sensitivity, the benefits for routine screening require further study especially in view of the frequent expression of ANA in a control population especially among females; in these preliminary studies, we have not addressed the differences in reactivity of males and females in the control population. We have also not assessed any differences with regard to the use of plasma or sera or the use of different blocking agents which potentially could contain nuclear material [68].

While ANA testing is a component of initial patient evaluation, repeat testing is increasingly performed to determine patient eligibility for clinical trials or utilization of certain medications [5,6]. Thus, belimumab, an anti-BAFF therapy, is approved for use in patients who are positive for ANA or anti-DNA. In determining the treatment of a patient with established disease, a more sensitive assay may be useful to determine continued ANA expression in patients whose sera may produce uncertain or negative results on assays that have been designed for purposes of diagnosis and therefore may be less sensitive.

Finally, with its utilization of NABP as an affinity matrix, these studies provide insight into the physical-chemical interactions of nuclear antigens emerging from dead and dying cells and suggest interconnectedness and attachment. This situation may not be surprising in view of the actual structure of a nucleus which is densely packed with highly interactive molecules. Future studies are therefore in progress to refine this approach as a platform for ANA testing as well as assessing the interactions of DNA and RNA with other molecules in the cell.

## Supporting Information

S1 Table ELISA of directly-coated or NABP-captured CT DNA, detected with SLE plasmas or no plasma. The table presents data from an ELISA shown in Fig 1 on the binding of SLE plasma and no plasma to calf thymus DNA either coated directly to a microtiter plate or coated to a plate pre-coated with an NABP.
(PDF)

S2 Table ELISA of directly-coated or PLL-capture nucleosomes, detected with SLE plasmas. The table presents data for the experiment in Fig 2 on the binding of plasma to nucleosomes either directly coated to microtiter plates or coated to plates that had been pre-coated with PLL.
(PDF)

S3 Table ELISA of directly-coated or PLL-captured STS-supernatant, detected with SLE plasmas. The table presents data for Fig 3 on binding of SLE plasma to a supernatant of staurosporine(STS)- treated Jurkat cells coated directly to a microtiter plate or coated to a plate pre-coated with PLL.
(PDF)

S4 Table ELISA of directly-coated or NABP-captured STS-supernatant, detected with index plasmas. The table presents data on the binding of index plasma to the STS supernatant as shown in Fig 4.
(PDF)

S5 Table ELISA of directly-coated or PLL-captured STS-supernatant or DNased STS supernatant, detected with SLE plasmas and index plasmas. The table presents data used to calculate results in Table 1 on the effects of DNase treatment on the extent of binding of SLE and index plasmas to the STS supernatant.
(PDF)

S6 Table ELISA of directly-coated or PLL-captured STS-supernatant treated with a range of RNase concentrations, detected with SLE plasma 1. The table presents data used in Table 1 to assess the effects of different concentrations of RNase on the binding of an SLE plasma to STS supernatant.
(PDF)

S7 Table ELISA of directly-coated or PLL-captured STS-supernatant, detected with a range of dilutions of index and normal plasmas. The table presents data on the binding of different index plasmas to STS supernatant either coated directly to a microtiter plate or a plate pre-coated with PLL. The data were used for Fig 6.
(PDF)

S8 Table ELISA of directly-coated or PLL-captured tetanus toxoid (tt), detected with SLE plasmas. The table presents data used for Fig 7 on the binding of plasmas to tetanus toxoid coated directly onto a microtiter plate or a plate pre-coated with PLL.
(PDF)

S9 Table Comparison of prototype ANA capture assay with BioPlex^®^ 2200 ANA assay data. The table presents data for Table 2 on the comparison of a prototype assay with the BioPlex^®^ 2200 assay.
(PDF)
