# Supplementary material for: The Use of Poly-L-Lysine as a Capture Agent to Enhance the Detection of Antinuclear Antibodies by ELISA
Source: PLoS One. 2016 Sep 9;11(9):e0161818. doi: 10.1371/journal.pone.0161818 (PMC5017613; doi:10.1371/journal.pone.0161818)
Supplement: S1 Table — The table presents data from an ELISA shown in Fig 1 on the binding of SLE plasma and no plasma to calf thymus DNA either coated directly to a microtiter plate or coated to a plate pre-coated with an NABP. (PDF) [file pone.0161818.s001.pdf]

Raw data for Figure 1

ELISA of directly-coated or NABP-captured CT DNA, detected with SLE Plasmas or no plasma

	SLE Plasma 1		SLE Plasma 2		SLE Plasma 3		No plasma	
CT DNA coated plate	OD_{450}		OD_{450}		OD_{450}		OD_{450}	
CT DNA (ng/ml)	well 1	well2	well 1	well 2	well 1	well 2	well 1	well 2
5,000	0.464	0.410	0.419	0.476	0.469	0.547	0.048	0.051
2,000	0.216	0.172	0.223	0.239	0.201	0.189	0.055	0.567*
1,000	0.171	0.133	0.156	0.206	0.172	0.168	0.047	0.099
500	0.092	0.086	0.087	0.126	0.126	0.085	0.055	0.048
250	0.076	0.085	0.085	0.076	0.072	0.075	0.049	0.050
100	0.068	0.067	0.065	0.070	0.103	0.067	0.048	0.053
50	0.067	0.065	0.072	0.063	0.082	0.092	0.049	0.059
25	0.059	0.062	0.065	0.056	0.092	0.125	0.051	0.050
10	0.061	0.064	0.067	0.060	0.054	0.070	0.061	0.049
0 (SSC buffer only)	0.057	0.068	0.064	0.057	0.054	0.055	0.049	0.050

PAMAM coated plate<br>CT DNA for Capture<br>(ng/ml)	well 1	well2	well 1	well 2	well 1	well 2	well 1	well 2
5,000	0.877	0.652	2.646	2.837	0.131	0.158	0.152	0.304
2,000	0.791	0.608	2.557	2.601	0.111	0.150	0.150	0.267
1,000	0.428	0.272	2.069	2.132	0.106	0.153	0.097	0.177
500	0.352	0.179	1.130	1.284	0.154	0.117	0.102	0.139
250	0.321	0.208	0.736	0.509	0.097	0.124	0.155	0.157
100	0.371	0.149	0.274	0.288	0.135	0.123	0.091	0.141
50	0.174	0.114	0.176	0.197	0.099	0.149	0.080	0.194
25	0.130	0.104	0.153	0.129	0.122	0.172	0.069	0.115
10	0.109	0.092	0.125	0.119	0.101	0.113	0.119	0.085
0 (PBS buffer only)	0.108	0.102	0.105	0.105	0.103	0.094	0.098	0.094

HDMBR coated plate<br>CT DNA for Capture<br>(ng/ml)	well 1	well2	well 1	well 2	well 1	well 2	well 1	well 2
5,000	0.160	0.133	0.545	0.894	0.156	0.108	0.106	0.081
2,000	0.246	0.193	0.597	0.603	0.127	0.122	0.079	0.140
1,000	0.180	0.106	0.394	0.829	0.141	0.125	0.075	0.078
500	0.121	0.096	0.101	0.663	0.192	0.095	0.069	0.079
250	0.240	0.127	0.433	0.205	0.120	0.108	0.136	0.126
100	0.117	0.093	0.130	0.199	0.142	0.100	0.070	0.084
50	0.095	0.080	0.082	0.132	0.089	0.162	0.066	0.120
25	0.089	0.075	0.076	0.073	0.137	0.145	0.065	0.077
10	0.077	0.069	0.067	0.080	0.074	0.093	0.088	0.069
0 (PBS buffer only)	0.070	0.071	0.076	0.071	0.081	0.076	0.083	0.070

Poly-l-lysine coated plate<br>CT DNA for Capture<br>(ng/ml)	well 1	well2	well 1	well 2	well 1	well 2	well 1	well 2
5,000	0.825	1.141	2.944	2.961	0.192	0.164	0.075	0.059
2,000	0.322	0.336	1.986	2.000	0.142	0.115	0.078	0.081
1,000	0.194	0.195	1.019	1.022	0.112	0.100	0.079	0.083
500	0.126	0.138	0.410	0.514	0.114	0.093	0.079	0.077
250	0.109	0.128	0.312	0.254	0.101	0.088	0.128	0.072
100	0.114	0.111	0.175	0.184	0.111	0.095	0.086	0.077
50	0.106	0.116	0.135	0.165	0.105	0.097	0.087	0.068
25	0.103	0.112	0.118	0.118	0.098	0.093	0.084	0.081
10	0.102	0.100	0.106	0.123	0.100	0.091	0.095	0.074
0 (PBS buffer only)	0.101	0.104	0.119	0.114	0.092	0.093	0.086	0.069

Protamine sulfate coated plate<br>CT DNA for Capture<br>(ng/ml)	well 1	well2	well 1	well 2	well 1	well 2	well 1	well 2
5,000	0.658	0.736	2.773	2.771	0.122	0.122	0.060	0.065
2,000	0.503	0.512	2.592	2.625	0.128	0.116	0.060	0.102
1,000	0.274	0.295	1.855	1.978	0.142	0.116	0.070	0.070
500	0.217	0.218	1.070	1.258	0.103	0.135	0.066	0.086
250	0.197	0.201	0.815	0.833	0.133	0.121	0.061	0.077
100	0.186	0.190	0.568	0.564	0.107	0.121	0.073	0.083
50	0.177	0.187	0.410	0.448	0.123	0.109	0.075	0.088
25	0.171	0.163	0.329	0.345	0.099	0.109	0.077	0.075
10	0.168	0.149	0.275	0.294	0.119	0.110	0.072	0.085
0 (PBS buffer only)	0.154	0.157	0.214	0.224	0.119	0.120	0.063	0.076

* outlier; datum not used
