# Supplementary material for: The Use of Poly-L-Lysine as a Capture Agent to Enhance the Detection of Antinuclear Antibodies by ELISA
Source: PLoS One. 2016 Sep 9;11(9):e0161818. doi: 10.1371/journal.pone.0161818 (PMC5017613; doi:10.1371/journal.pone.0161818)
Supplement: S2 Table — The table presents data for the experiment in Fig 2 on the binding of plasma to nucleosomes either directly coated to microtiter plates or coated to plates that had been pre-coated with PLL. (PDF) [file pone.0161818.s002.pdf]

Raw data for Figure 2

ELISA of directly-coated or PLL-capture nucleosomes, detected with SLE Plasmas

	SLE Plasma 1		SLE Plalsma 2		SLE Plasma 3	
nucleosome coated plate	OD_{450}		OD_{450}		OD_{450}	
nucleosome (DNA) (ng/ml)	well 1	well2	well 1	well 2	well 1	well 2
5,000	1.485	1.883	2.085	2.286	0.452	0.477
2,000	1.080	1.416	1.914	1.874	0.384	0.437
1,000	0.625	0.844	1.190	1.108	0.336	0.354
500	0.227	0.314	0.269	0.253	0.325	0.303
250	0.207	0.239	0.161	0.144	0.230	0.278
100	0.200	0.182	0.113	0.124	0.272	0.279
50	0.162	0.139	0.113	0.104	0.186	0.245
25	0.127	0.118	0.087	0.089	0.218	0.238
10	0.091	0.090	0.083	0.079	0.132	0.198
0 (20mM Tris pH 8, 150 mM Nacl)	0.074	0.072	0.070	0.060	0.127	0.122

Poly-l-lysine coated plate						
nucleosome (DNA) for capture (ng/ml)	well 1	well2	well 1	well 2	well 1	well 2
5,000	3.068	3.100	3.299	3.285	0.523	0.510
2,000	2.883	2.963	3.301	3.151	0.369	0.373
1,000	2.366	2.310	2.665	2.728	0.305	0.287
500	1.286	1.352	1.664	1.587	0.270	0.193
250	0.518	0.677	0.490	0.621	0.181	0.181
100	0.308	0.321	0.261	0.216	0.266	0.178
50	0.179	0.225	0.147	0.145	0.196	0.228
25	0.147	0.159	0.127	0.115	0.211	0.246
10	0.129	0.142	0.123	0.114	0.170	0.184
0 (ELISA Dilution Buffer)	0.135	0.147	0.130	0.124	0.191	0.179
