# Supplementary material for: The Use of Poly-L-Lysine as a Capture Agent to Enhance the Detection of Antinuclear Antibodies by ELISA
Source: PLoS One. 2016 Sep 9;11(9):e0161818. doi: 10.1371/journal.pone.0161818 (PMC5017613; doi:10.1371/journal.pone.0161818)
Supplement: S3 Table — The table presents data for Fig 3 on binding of SLE plasma to a supernatant of staurosporine(STS)- treated Jurkat cells coated directly to a microtiter plate or coated to a plate pre-coated with PLL. (PDF) [file pone.0161818.s003.pdf]

Raw data for Figure 3

ELISA of directly-coated or PLL-captured STS-supernatant, detected with SLE Plasmas

STS-supernatant coated plate	SLE Plasma 1 OD$_{450}$		SLE Plasma 2 OD$_{450}$		SLE Plasma 3 OD$_{450}$	
STS-supernatant (DNA) (ng/ml)	well 1	well2	well 1	well 2	well 1	well 2
2,000	0.551	0.591	0.671	0.668	0.715	0.704
1,000	0.537	0.560	0.721	0.699	0.736	0.652
500	0.526	0.558	0.627	0.605	0.675	0.612
250	0.502	0.521	0.610	0.588	0.563	0.597
100	0.424	0.433	0.543	0.556	0.485	0.496
50	0.333	0.345	0.450	0.468	0.391	0.374
25	0.222	0.232	0.306	0.301	0.260	0.250
10	0.140	0.131	0.154	0.158	0.142	0.149
5	0.090	0.091	0.101	0.096	0.105	0.102
2.5	0.080	0.084	0.088	0.079	0.099	0.087
1	0.064	0.064	0.090	0.065	0.073	0.070
0 (PBS)	0.058	0.065	0.066	0.057	0.062	0.063

Poly-l-lysine coated plate						
STS-supernatant (DNA) for capture (ng/ml)	well 1	well2	well 1	well 2	well 1	well 2
2,000	2.691	2.759	2.823	2.885	2.820	2.820
1,000	2.469	2.440	2.495	2.471	2.133	1.978
500	1.394	1.463	0.911	1.013	0.879	0.776
250	0.442	0.481	0.374	0.377	0.323	0.302
100	0.210	0.260	0.248	0.234	0.192	0.204
50	0.159	0.198	0.174	0.171	0.175	0.147
25	0.148	0.188	0.167	0.134	0.145	0.139
10	0.129	0.153	0.141	0.117	0.152	0.137
5	0.121	0.146	0.125	0.106	0.140	0.142
2.5	0.117	0.151	0.121	0.110	0.140	0.151
1	0.110	0.127	0.234	0.111	0.139	0.145
0 (ELISA Dilution Buffer)	0.106	0.109	0.105	0.106	0.131	0.147
