# Supplementary material for: The Use of Poly-L-Lysine as a Capture Agent to Enhance the Detection of Antinuclear Antibodies by ELISA
Source: PLoS One. 2016 Sep 9;11(9):e0161818. doi: 10.1371/journal.pone.0161818 (PMC5017613; doi:10.1371/journal.pone.0161818)
Supplement: S4 Table — The table presents data on the binding of index plasma to the STS supernatant as shown in Fig 4. (PDF) [file pone.0161818.s004.pdf]

Raw data for Figure 4

ELISA of directly-coated or NABP-captured STS-supernatant, detected with index plasmas

	anti-dsDNA plasma	anti-SSA plasma	anti-SSB plasma	
STS-supernatant coated plate	OD_{450}	OD_{450}	OD_{450}	
STS-supernatant (DNA) (ng/ml)			well 1	well 2
2,000	0.505	0.757	1.075	0.977
1,500	0.455	0.616	1.029	0.928
1,000	0.437	0.623	0.939	0.895
500	0.395	0.555	0.908	0.836
250	0.346	0.466	0.829	0.797
0	0.060	0.108	0.074	0.073
500ng/ml Poly-l-lysine coated plate				
STS-supernatant (DNA) for capture (ng/ml)			well 1	well 2
2,000	1.961	1.433	2.245	2.239
1,500	1.440	1.183	2.110	2.081
1,000	1.351	1.203	1.885	1.842
500	0.825	1.112	1.323	1.298
250	0.630	0.700	0.817	0.750
0	0.132	0.198	0.240	0.255

	anti-RNP plasma		anti-Histone plasma		anti-Sm plasma	
STS-supernatant coated plate	OD_{450}		OD_{450}		OD_{450}	
STS-supernatant (DNA) (ng/ml)	well 1	well 2	well 1	well 2	well 1	well 2
1,000	2.574	2.597	2.664	2.619	2.035	2.093
500	2.249	2.333	2.591	2.447	2.150	2.094
250	2.247	2.368	2.659	2.511	1.830	1.712
100	2.371	2.115	2.276	2.158	1.433	1.220
50	1.087	1.115	1.665	1.506	0.894	0.800
25	0.429	0.361	0.877	0.806	0.426	0.433
10	0.132	0.160	0.424	0.473	0.137	0.154
5	0.105	0.104	0.326	0.262	0.100	0.073
2.5	0.066	0.072	0.157	0.170	0.085	0.070
1	0.084	0.069	0.138	0.158	0.061	0.087
0	0.069	0.060	0.062	0.076	0.065	0.064
500ng/ml Poly-l-lysine coated plate						
STS-supernatant (DNA) for capture (ng/ml)	well 1	well 2	well 1	well 2	well 1	well 2
1,000	3.240	3.320	3.396	3.356	1.457	1.660
500	3.008	3.176	3.321	3.328	1.955	1.937
250	2.557	2.744	3.306	3.117	1.690	1.562
100	2.055	2.091	2.960	2.629	1.012	1.071
50	1.459	1.632	2.457	2.355	0.534	0.624
25	1.057	1.143	1.897	1.789	0.327	0.283
10	0.518	0.562	1.207	1.089	0.195	0.193
5	0.290	0.346	0.907	0.815	0.148	0.165
2.5	0.237	0.259	0.689	0.725	0.132	0.155
1	0.153	0.152	0.674	0.653	0.111	0.135
0	0.136	0.115	0.605	0.645	0.133	0.127
