# Supplementary material for: The Use of Poly-L-Lysine as a Capture Agent to Enhance the Detection of Antinuclear Antibodies by ELISA
Source: PLoS One. 2016 Sep 9;11(9):e0161818. doi: 10.1371/journal.pone.0161818 (PMC5017613; doi:10.1371/journal.pone.0161818)
Supplement: S5 Table — The table presents data used to calculate results in Table 1 on the effects of DNase treatment on the extent of binding of SLE and index plasmas to the STS supernatant. (PDF) [file pone.0161818.s005.pdf]

Raw Data for Table 1

ELISA of directly-coated or PLL-captured STS-supernatant or DNased STS supernatant, detected with SLE Plasmas and index plasmas.

	exp. 1	exp. 2		
	SLE plasma 1	SLE plasma 1		
direct coat No DNase	OD_{450}	OD_{450}		
STS sup ng/ml		well 1	well 2	ave
2,000	0.769	0.715	0.636	0.676
1,500	0.672	0.582	0.547	0.565
1,000	0.644	0.531	0.531	0.531
500	0.518	0.427	0.467	0.447
250	0.499	0.396	0.397	0.397
0	0.062	0.102	0.082	0.092

direct coat DNased	SLE plasma 1	SLE plasma 1		
STS sup ng/ml		well 1	well 2	ave
2,000	0.781	0.690	0.644	0.667
1,500	0.579	0.593	0.589	0.591
1,000	0.593	0.487	0.516	0.502
500	0.505	0.428	0.397	0.413
250	0.447	0.328	0.332	0.330
0	0.069	0.088	0.081	0.085

PLL captured	SLE plasma 1	SLE plasma 1		
No DNase STS sup ng/ml		well 1	well 2	ave
2,000	3.217	3.036	2.924	2.980
1,500	2.340	2.767	2.960	2.864
1,000	2.966	2.647	2.780	2.714
500	2.699	1.961	1.995	1.978
250	1.609	0.765	0.860	0.813
0	0.148	0.139	0.182	0.161

PLL captured	SLE plasma 1	SLE plasma 1		
DNased STS sup ng/ml		well 1	well 2	ave
2,000	2.792	2.527	2.478	2.503
1,500	2.242	2.252	2.339	2.296
1,000	2.185	1.847	1.902	1.875
500	1.441	0.996	1.082	1.039
250	0.566	0.410	0.424	0.417
0	0.160	0.151	0.172	0.162

	exp. 1	exp. 2		
	SLE plasma 2	SLE plasma 2		
direct coat No DNase	OD_{450}	OD_{450}		
STS sup ng/ml		well 1	well 2	ave
2,000	0.820	0.715	0.672	0.694
1,500	0.791	0.600	0.616	0.608
1,000	0.791	0.605	0.600	0.603
500	0.705	0.585	0.581	0.583
250	0.651	0.550	0.561	0.556
0	0.078	0.100	0.072	0.086

direct coat DNased	SLE plasma 2	SLE plasma 2		
STS sup ng/ml		well 1	well 2	ave
2,000	0.607	0.453	0.454	0.454
1,500	0.541	0.472	0.431	0.452
1,000	0.533	0.432	0.427	0.430
500	0.488	0.390	0.384	0.387
250	0.452	0.364	0.341	0.353
0	0.071	0.068	0.068	0.068

PLL captured	SLE plasma 2	SLE plasma 2		
No DNase STS sup ng/ml		well 1	well 2	ave
2,000	3.242	3.110	3.062	3.086
1,500	3.036	2.853	2.886	2.870
1,000	3.086	2.764	2.822	2.793
500	2.552	1.860	1.941	1.901
250	1.174	0.678	0.696	0.687
0	0.148	0.175	0.124	0.150

PLL captured	SLE plasma 2	SLE plasma 2		
DNased STS sup ng/ml		well 1	well 2	ave
2,000	2.629	2.337	2.378	2.358
1,500	1.764	2.006	2.051	2.029
1,000	1.667	1.317	1.408	1.363
500	0.597	0.465	0.516	0.491
250	0.294	0.217	0.216	0.217
0	0.159	0.125	0.154	0.140

	exp. 3		
	SLE plasma 2		
PLL captured	OD_{450}		
No DNase STS sup ng/ml	well 1	well 2	ave
1,000	3.053	3.013	3.033
500	2.516	2.415	2.466
250	1.023	0.991	1.007
100	0.454	0.402	0.428
50	0.291	0.265	0.278
25	0.226	0.197	0.212
10	0.181	0.187	0.184
5	0.175	0.172	0.174
2.5	0.160	0.147	0.154
1	0.223	0.145	0.184
0	0.143	0.142	0.143

PLL coated	SLE plasma 2		
DNased sup ng/ml	well 1	well 2	ave
1,000	1.270	1.266	1.268
500	0.597	0.598	0.598
250	0.289	0.264	0.277
100	0.182	0.177	0.180
50	0.194	0.150	0.172
25	0.188	0.144	0.166
10	0.152	0.143	0.148
5	0.164	0.141	0.153
2.5	0.162	0.131	0.147
1	0.205	0.136	0.171
0	0.155	0.159	0.157

	exp. 1	exp. 2			exp. 1
	SLE plasma 3	SLE plasma 3			anti-SSA
direct coat No DNase	OD_{450}		OD_{450}		OD_{450}
STS sup ng/ml		well 1	well 2	ave	
2,000	0.485	0.389	0.388	0.389	0.757
1,500	0.421	0.372	0.374	0.373	0.616
1,000	0.398	0.342	0.361	0.352	0.623
500	0.350	0.302	0.304	0.303	0.555
250	0.288	0.268	0.267	0.268	0.466
0	0.075	0.076	0.076	0.076	0.108
direct coat DNased	SLE plasma 3		SLE plasma 3		anti-SSA
STS sup ng/ml		well 1	well 2	ave	
2,000	0.409	0.370	0.356	0.363	0.645
1,500	0.326	0.334	0.318	0.326	0.531
1,000	0.326	0.306	0.309	0.308	0.546
500	0.274	0.264	0.255	0.260	0.479
250	0.279	0.229	0.258	0.244	0.494
0	0.065	0.069	0.061	0.065	0.070
PLL captured	SLE plasma 3		SLE plasma 3		anti-SSA
No DNase STS sup ng/ml		well 1	well 2	ave	
2,000	2.753	2.407	2.553	2.480	1.433
1,500	2.432	2.311	2.213	2.262	1.183
1,000	2.318	2.024	1.945	1.985	1.203
500	1.346	0.816	0.925	0.871	1.112
250	0.494	0.308	0.297	0.303	0.700
0	0.135	0.187	0.148	0.168	0.198
PLL captured	SLE plasma 3		SLE plasma 3		anti-SSA
DNased STS sup ng/ml		well 1	well 2	ave	
2,000	1.924	1.899	1.904	1.902	2.041
1,500	1.271	1.635	1.631	1.633	1.587
1,000	1.258	1.179	1.099	1.139	1.694
500	0.493	0.438	0.503	0.471	1.070
250	0.287	0.204	0.203	0.204	0.660
0	0.196	0.161	0.135	0.148	0.145

	exp. 1		exp. 2		exp. 1		exp. 2	
	anti-SSB		anti-SSB		anti-dsDNA		anti-dsDNA	
direct coat No DNase	OD_{450}		OD_{450}		OD_{450}		OD_{450}	
STS sup ng/ml		well 1	well 2	ave		well 1	well 2	ave
2,000	1.237	1.075	0.977	1.026	0.505	0.474	0.555	0.515
1,500	1.037	1.029	0.928	0.979	0.455	0.419	0.442	0.431
1,000	0.998	0.939	0.895	0.917	0.437	0.424	0.394	0.409
500	0.944	0.908	0.836	0.872	0.395	0.362	0.374	0.368
250	0.849	0.829	0.797	0.813	0.346	0.306	0.317	0.312
0	0.063	0.074	0.073	0.074	0.060	0.073	0.071	0.072
direct coat DNased	anti-SSB		anti-SSB		anti-dsDNA		anti-dsDNA	
STS sup ng/ml		well 1	well 2	ave		well 1	well 2	ave
2,000	1.153	0.952	0.877	0.915	0.488	0.422	0.448	0.435
1,500	0.933	0.873	0.812	0.843	0.394	0.383	0.372	0.378
1,000	0.939	0.810	0.761	0.786	0.388	0.386	0.346	0.366
500	0.849	0.734	0.658	0.696	0.333	0.331	0.324	0.328
250	0.900	0.600	0.641	0.621	0.303	0.302	0.319	0.311
0	0.075	0.069	0.062	0.066	0.062	0.061	0.069	0.065
PLL captured	anti-SSB		anti-SSB		anti-dsDNA		anti-dsDNA	
No DNase STS sup ng/ml		well 1	well 2	ave		well 1	well 2	ave
2,000	2.621	2.245	2.239	2.242	1.961	1.840	1.754	1.797
1,500	2.245	2.110	2.081	2.096	1.440	1.514	1.617	1.566
1,000	2.049	1.885	1.842	1.864	1.351	0.999	1.110	1.055
500	1.599	1.323	1.298	1.311	0.825	0.483	0.492	0.488
250	0.878	0.817	0.750	0.784	0.630	0.246	0.253	0.250
0	0.249	0.240	0.255	0.248	0.132	0.125	0.109	0.117
PLL captured	anti-SSB		anti-SSB		anti-dsDNA		anti-dsDNA	
DNased STS sup ng/ml		well 1	well 2	ave		well 1	well 2	ave
2,000	1.765	1.707	1.725	1.716	1.093	0.931	0.931	0.931
1,500	0.956	1.404	1.453	1.429	0.635	0.619	0.619	0.619
1,000	0.886	1.072	1.089	1.081	0.571	0.424	0.424	0.424
500	0.523	0.628	0.524	0.576	0.508	0.188	0.188	0.188
250	0.351	0.259	0.321	0.290	0.425	0.204	0.204	0.204
0	0.515	0.230	0.260	0.245	0.139	0.133	0.133	0.133

	exp. 1		exp. 2	
	anti-histone		anti-histone	
direct coat No DNase	OD_{450}		OD_{450}	
STS sup ng/ml		well 1	well 2	ave
2,000	2.748	2.704	2.845	2.775
1,500	2.570	2.610	2.437	2.524
1,000	2.571	2.405	2.452	2.429
500	2.447	2.274	2.295	2.285
250	2.162	2.159	2.269	2.214
0	0.105	0.072	0.078	0.075
direct coat DNased	anti-histone		anti-histone	
STS sup ng/ml		well 1	well 2	ave
2,000	2.426	2.468	2.473	2.471
1,500	2.444	2.189	2.337	2.263
1,000	2.523	2.246	2.265	2.256
500	2.179	2.149	2.195	2.172
250	2.134	1.839	1.939	1.889
0	0.107	0.065	0.062	0.064
PLL captured			anti-histone	
No DNase STS sup ng/ml	anti-histone	well 1	well 2	ave
2,000	3.302	3.265	3.335	3.300
1,500	3.304	3.270	3.229	3.250
1,000	3.290	3.144	3.217	3.181
500	3.242	3.068	3.135	3.102
250	3.143	2.722	2.843	2.783
0	0.530	0.451	0.411	0.431
PLL captured	anti-histone		anti-histone	
DNased STS sup ng/ml		well 1	well 2	ave
2,000	3.298	3.350	3.287	3.319
1,500	3.258	3.286	3.144	3.215
1,000	3.236	3.129	3.018	3.074
500	3.272	3.006	2.484	2.745
250	3.049	2.893	2.897	2.895
0	0.528	0.502	0.499	0.501

		exp. 3	
		anti-histone	
PLL captured		OD_{450}	
No DNase STS sup ng/ml	well 1	well 2	ave
1,000	3.396	3.356	3.376
500	3.321	3.328	3.325
250	3.306	3.117	3.212
100	2.960	2.629	2.795
50	2.457	2.355	2.406
25	1.897	1.789	1.843
10	1.207	1.089	1.148
5	0.907	0.815	0.861
2.5	0.689	0.725	0.707
1	0.674	0.653	0.664
0	0.605	0.645	0.625
PLL coated		anti-histone	
DNased sup ng/ml	well 1	well 2	ave
1,000	3.338	3.369	3.354
500	3.364	3.266	3.315
250	3.146	3.060	3.103
100	2.700	2.543	2.622
50	2.074	1.950	2.012
25	1.435	1.312	1.374
10	0.979	0.852	0.916
5	0.802	0.684	0.743
2.5	0.624	0.623	0.624
1	0.633	0.603	0.618
0	0.621	0.606	0.614

	exp. 1	exp. 1
	anti-RNP	anti-Sm
direct coat No DNase	OD_{450}	OD_{450}
STS sup ng/ml		
2,000	2.301	2.336
1,500	2.217	2.565
1,000	2.574	2.161
500	1.885	1.959
250	1.902	1.704
0	0.060	0.066
direct coat DNased	anti-RNP	anti-Sm
STS sup ng/ml		
2,000	2.577	2.337
1,500	2.279	2.431
1,000	2.012	1.917
500	1.900	1.950
250	1.778	1.730
0	0.057	0.055
PLL captured	anti-RNP	anti-Sm
No DNase STS sup ng/ml		
2,000	3.219	2.570
1,500	3.280	1.982
1,000	3.274	2.033
500	2.979	1.871
250	2.793	1.764
0	0.140	0.152
PLL captured	anti-RNP	anti-Sm
DNased STS sup ng/ml		
2,000	3.002	2.809
1,500	2.742	2.405
1,000	2.638	2.192
500	2.579	2.259
250	2.196	2.079
0	0.126	0.120

		exp. 3			exp. 3	
		anti-RNP			anti-Sm	
PLL captured		OD_{450}			OD_{450}	
No DNase STS sup ng/ml	well 1	well2	ave	well 1	well 2	ave
1,000	3.240	3.320	3.280	1.457	1.660	1.559
500	3.008	3.176	3.092	1.955	1.937	1.946
250	2.557	2.744	2.651	1.690	1.562	1.626
100	2.055	2.091	2.073	1.012	1.071	1.042
50	1.459	1.632	1.546	0.534	0.624	0.579
25	1.057	1.143	1.100	0.327	0.283	0.305
10	0.518	0.562	0.540	0.195	0.193	0.194
5	0.290	0.346	0.318	0.148	0.165	0.157
2.5	0.237	0.259	0.248	0.132	0.155	0.144
1	0.153	0.152	0.153	0.111	0.135	0.123
0	0.136	0.115	0.126	0.133	0.127	0.130
PLL coated		anti-RNP			anti-Sm	
DNased sup ng/ml	well 1	well2	ave	well 1	well 2	ave
1,000	2.533	2.425	2.479	2.052	2.103	2.078
500	2.085	2.091	2.088	2.045	1.954	2.000
250	1.742	1.612	1.677	1.624	1.639	1.632
100	1.159	1.083	1.121	0.809	0.911	0.860
50	0.799	0.719	0.759	0.434	0.470	0.452
25	0.540	0.528	0.534	0.298	0.230	0.264
10	0.305	0.280	0.293	0.184	0.157	0.171
5	0.197	0.195	0.196	0.134	0.130	0.132
2.5	0.170	0.177	0.174	0.121	0.117	0.119
1	0.129	0.169	0.149	0.116	0.105	0.111
0	0.132	0.135	0.134	0.103	0.102	0.103
