# Supplementary material for: The Use of Poly-L-Lysine as a Capture Agent to Enhance the Detection of Antinuclear Antibodies by ELISA
Source: PLoS One. 2016 Sep 9;11(9):e0161818. doi: 10.1371/journal.pone.0161818 (PMC5017613; doi:10.1371/journal.pone.0161818)
Supplement: S6 Table — The table presents data used in Table 1 to assess the effects of different concentrations of RNase on the binding of an SLE plasma to STS supernatant. (PDF) [file pone.0161818.s006.pdf]

Raw data for Figure 5

ELISA of directly-coated or PLL-captured
STS-supernatant treated with a range
of RNase concentrations,
detected with SLE Plasma 1

RNase KU/ml	direct coat OD_{450}	PLL capture OD_{450}
0	0.509	2.904
0.015	0.473	1.949
0.075	0.453	1.203
0.15	0.441	0.846
0.75	0.440	0.446
1.5	0.496	0.410
