# Supplementary material for: The Use of Poly-L-Lysine as a Capture Agent to Enhance the Detection of Antinuclear Antibodies by ELISA
Source: PLoS One. 2016 Sep 9;11(9):e0161818. doi: 10.1371/journal.pone.0161818 (PMC5017613; doi:10.1371/journal.pone.0161818)
Supplement: S7 Table — The table presents data on the binding of different index plasmas to STS supernatant either coated directly to a microtiter plate or a plate pre-coated with PLL. The data were used for Fig 6. (PDF) [file pone.0161818.s007.pdf]

Raw data for Figure 6

ELISA of directly-coated or PLL-captured STS-supernatant, detected with a range of dilutions of index and normal plasmas

Direct coat STS-supernatant (OD_{450})

Plasma dilution	anti-dsDNA plasma	anti-histone plasma	anti-RNP plasma	anti-SSA plasma	anti-SSB plasma	normal 6 plasma	normal 7 plasma	normal 1 plasma
1/800	0.547	2.423	2.234	0.772	1.146	0.504	0.959	0.571
1/1,600	0.383	1.959	2.026	0.717	0.935	0.375	0.619	0.429
1/3,200	0.312	1.382	1.841	0.495	0.596	0.275	0.414	0.334
1/6,400	0.232	0.903	1.432	0.406	0.443	0.241	0.310	0.326
1/12,800	0.210	0.579	1.107	0.380	0.337	0.234	0.239	0.268
1/25,600	0.204	0.370	0.768	0.354	0.268	0.249	0.243	0.239

PLL Capture STS-supernatant (OD_{450})

Plasma dilution	anti-dsDNA plasma	anti-histone plasma	anti-RNP plasma	anti-SSA plasma	anti-SSB plasma	normal 6 plasma	normal 7 plasma	normal 1 plasma
1/800	1.193	3.093	3.190	1.507	2.422	0.479	0.977	1.428
1/1,600	0.821	3.028	2.942	1.318	1.867	0.342	0.546	0.909
1/3,200	0.483	2.857	2.666	1.118	1.186	0.194	0.330	0.539
1/6,400	0.259	2.283	2.429	0.979	0.708	0.167	0.218	0.353
1/12,800	0.183	1.834	1.926	0.841	0.403	0.142	0.149	0.229
1/25,600	0.144	1.073	1.356	0.624	0.288	0.107	0.121	0.169
