# Supplementary material for: The Use of Poly-L-Lysine as a Capture Agent to Enhance the Detection of Antinuclear Antibodies by ELISA
Source: PLoS One. 2016 Sep 9;11(9):e0161818. doi: 10.1371/journal.pone.0161818 (PMC5017613; doi:10.1371/journal.pone.0161818)
Supplement: S8 Table — The table presents data used for Fig 7 on the binding of plasmas to tetanus toxoid coated directly onto a microtiter plate or a plate pre-coated with PLL. (PDF) [file pone.0161818.s008.pdf]

Raw data for Figure 7

ELISA of directly-coated or PLL-captured tetanus toxoid (tt), detected with SLE Plasmas

tetanus toxoid ng/ml	tt direct coat SLE Plasma 1 OD_{450}	tt PLL captured SLE Plasma 1 OD_{450}	tt direct coat SLE Plasma 2 OD_{450}	tt PLL captured SLE Plasma 2 OD_{450}	tt direct coat SLE Plasma 3 OD_{450}	tt PLL captured SLE Plasma 3 OD_{450}
500	0.263	0.085	0.864	0.078	0.950	0.090
250	0.183	0.077	0.769	0.087	0.901	0.089
100	0.106	0.085	0.423	0.076	0.641	0.086
50	0.065	0.077	0.253	0.076	0.334	0.084
25	0.065	0.081	0.154	0.073	0.192	0.085
10	0.056	0.078	0.102	0.084	0.100	0.079
5	0.051	0.076	0.070	0.076	0.079	0.086
2.5	0.061	0.087	0.054	0.075	0.056	0.087
1	0.052	0.079	0.056	0.078	0.053	0.083
0.5	0.053	0.082	0.059	0.095	0.053	0.086
0.25	0.053	0.081	0.059	0.094	0.053	0.079
0	0.051	0.083	0.051	0.079	0.048	0.078
