# Supplementary material for: The Use of Poly-L-Lysine as a Capture Agent to Enhance the Detection of Antinuclear Antibodies by ELISA
Source: PLoS One. 2016 Sep 9;11(9):e0161818. doi: 10.1371/journal.pone.0161818 (PMC5017613; doi:10.1371/journal.pone.0161818)
Supplement: S9 Table — The table presents data for Table 2 on the comparison of a prototype assay with the BioPlex® 2200 assay. (PDF) [file pone.0161818.s009.pdf]

Data for Table2. Comparison of prototype ANA capture assay with BioPlex® 2200 ANA assay data.

		BioPlex®® 2200 ANA Screen Results									
	Capture ANA-EIA ANA #	dsDNA	Chrom	Ribo-P	Sm	SmRNP	RNP A	RNP 68	SSB	SSA-52	SSA-60
Cut-off	3.9	10 IU	1 AI	1 AI	1 AI	1 AI	1 AI	1 AI	1 AI	1 AI	1 AI
Sample ID:											
0011	1.90	0.0	0.6	1.0	0.0	6.6	1.8	0.0	0.0	0.8	84.7
0014	32.30	19.8	141.9	0.2	379.8	352.3	2.2	0.6	0.2	0.2	1.0
0033	100.50	180.6	9.6	9.0	4.2	778.7	67.3	0.6	0.0	0.0	0.0
0046	24.30	1.0	96.9	0.2	104.5	148.5	0.8	0.8	268.6	0.6	313.7
0050	392.00	95.2	1050.6	0.6	3547.7	5054.9	78.8	130.7	0.4	0.0	0.6
0119	17.85	17.2	4.4	0.2	11.6	10.6	4.8	0.4	0.0	0.0	0.0
0148	0.11	0.4	0.0	0.0	0.0	0.0	0.2	0.0	0.0	0.0	0.0
0167	35.85	0.4	9.4	0.0	0.6	609.7	5.0	2.0	0.0	0.0	0.0
0192	37.85	54.4	1176.6	0.2	0.0	0.6	1.0	0.0	0.0	0.0	12.6
0216	1.78	0.2	0.2	0.0	0.0	0.0	0.4	0.6	0.0	0.0	53.6
0221	0.82	2.2	0.2	0.0	0.0	2.6	0.6	0.0	0.0	0.0	0.0
0256	48.50	20.6	1.4	0.0	0.0	1.6	1.8	0.0	0.8	9.2	233.1
0259	15.80	26.8	4.2	0.2	11.0	10.4	4.8	0.4	0.0	0.0	0.0
0272	578.00	1.8	1298.5	4165.3	3278.5	3906.7	141.5	7.2	0.4	0.0	1.8
0295	646.00	1.2	398.7	5331.0	1219.3	1254.7	100.7	2.2	0.2	0.0	6.6
0405	4.07	3.4	0.4	0.0	0.2	1.4	1.4	0.0	0.0	0.0	0.6
0422	0.28	0.4	0.0	0.0	0.0	0.0	0.8	0.0	0.0	0.0	0.0
0428	17.35	5.2	5.4	2.4	0.2	100.5	5.8	0.2	0.0	8.4	1.0
0456	150.00	376.2	733.0	1.0	4070.1	1279.1	72.4	0.0	0.4	0.2	9.2
0477	11.45	3.2	3.2	0.0	63.6	28.4	0.6	1.8	0.0	0.0	0.0
0503	0.80	14.4	0.0	0.0	0.0	0.0	0.0	0.0	0.0	0.0	0.0
0510	1.03	5.0	0.2	0.0	0.0	0.0	2.4	0.0	0.0	0.0	0.0
0512	1.05	5.6	0.2	0.0	0.0	0.0	0.2	0.0	0.4	0.0	0.0
0534	0.55	0.6	0.0	0.0	0.0	0.0	0.6	0.2	0.0	0.0	0.0
0561	14.75	77.4	10.6	0.0	1.2	0.6	0.0	0.0	0.0	0.0	0.0
0576	1.23	263.6	0.6	0.0	0.0	0.0	0.2	0.0	0.2	0.0	0.2
0582	614.50	63.0	77.2	12347.6	227.1	56.0	1.6	0.0	0.2	0.0	2.2
0592	8.80	3.1	0.4	0.3	0.1	0.1	0.1	0.0	0.9	12.7	297.5
0619	486.50	212.2	981.9	98.4	7.6	14441.0	12.4	940.6	0.2	0.0	0.4
0640	2.12	36.2	1.4	0.0	0.0	0.0	0.0	0.0	0.0	0.0	0.0
0709	0.93	0.8	0.0	0.0	0.0	0.0	1.4	0.0	0.0	0.0	0.0
0729	1.12	1.8	0.0	0.0	0.0	0.0	1.4	0.0	0.0	0.0	0.0
0735	1.63	17.4	1.2	0.0	0.0	0.0	2.2	0.0	0.0	0.0	0.0
0750	10.05	0.2	1.8	0.0	0.2	126.1	0.6	0.6	0.2	0.0	0.2
0784	5.77	22.6	4.4	4.6	4.8	3.8	1.4	0.0	0.2	0.0	0.0
0836	0.88	1.6	0.0	0.0	0.0	0.2	0.2	0.0	0.2	0.0	0.0
0935	0.76	0.0	0.0	0.0	0.0	0.0	0.2	0.0	0.2	0.0	0.0
1020	576.50	182.2	1068.0	79.7	4.2	14368.0	109.6	690.4	0.2	0.0	8.6
1028	16.05	21.8	4.0	0.2	0.6	132.0	2.0	12.8	0.0	0.0	0.0
1076	0.12	0.4	0.0	0.0	0.0	0.0	0.0	0.0	0.4	0.0	0.0
1080	0.96	6.8	0.4	0.0	0.2	0.0	0.2	0.0	0.2	0.0	2.0
1309	154.00	1317.7	881.5	105.6	1235.2	798.5	8.6	0.0	1.6	0.8	59.7
1479	233.00	0.4	357.5	0.2	431.8	4277.0	0.4	145.8	0.2	0.0	0.2
1679	44.15	1.0	48.5	0.0	110.9	430.5	49.9	6.0	0.0	0.0	0.2
1742	11.25	0.6	0.2	0.1	0.2	0.1	0.0	0.0	281.5	6.5	304.3
1764	1.00	0.2	0.0	0.0	0.0	0.0	0.0	0.0	0.0	0.0	0.0
1787	1.03	2.0	0.2	0.0	0.0	0.0	2.6	0.0	0.0	0.8	14.2
1842	35.15	167.2	78.2	5.8	8.2	3.6	6.4	0.0	0.4	98.0	10.2
